# Evaluation of Whole-Genome Sequence, Genetic Diversity, and Agronomic Traits of Basmati Rice (*Oryza sativa* L.)

**DOI:** 10.3389/fgene.2020.00086

**Published:** 2020-02-21

**Authors:** D.S. Kishor, Jeonghwan Seo, Joong Hyoun Chin, Hee-Jong Koh

**Affiliations:** ^1^ Department of Plant Science, Plant Genomics and Breeding Institute, and Research Institute of Agriculture and Life Science, Seoul National University, Seoul, South Korea; ^2^ Department of Integrative Bio-industrial Engineering, Sejong University, Seoul, South Korea

**Keywords:** Basmati rice, aromatic, SNPs, NGS, gene ontology

## Abstract

Basmati is considered a unique varietal group of rice (*Oryza sativa* L.) because of its aroma and superior grain quality. Previous genetic analyses of rice showed that most of the Basmati varieties are classified into the *aromatic* group. Despite various efforts, genomic relationship of Basmati rice with other varietal groups and genomic variation in Basmati rice are yet to be understood. In the present study, we resequenced the whole genome of three traditional Basmati varieties at a coverage of more than 25X using Illumina HiSeq2500 and mapped the obtained sequences to the reference genome sequences of Nipponbare (*japonica* rice), Kasalath (*aus* rice), and Zhenshan 97 (*indica* rice). Comparison of these sequences revealed common single nucleotide polymorphisms (SNPs) in the genic regions of three Basmati varieties. Analysis of these SNPs revealed that Basmati varieties showed fewer sequence variations compared with the *aus* group than with the *japonica* and *indica* groups. Gene ontology (GO) enrichment analysis indicated that SNPs were present in genes with various biological, molecular, and cellular functions. Additionally, functional annotation of the Basmati mutated gene cluster shared by Nipponbare, Kasalath, and Zhenshan 97 was found to be associated with the metabolic process involved in the cellular aromatic compound, suggesting that aroma is an important specific genomic feature of Basmati varieties. Furthermore, 30 traditional Basmati varieties were classified into three different groups, *aromatic* (22 varieties), *aus* (four varieties), and *indica* (four varieties), based on genome-wide SNPs. All 22 *aromatic* Basmati varieties harbored the fragrant-inducing *Badh2* allele. We also performed comparative analysis of 13 key agronomic and grain quality traits of Basmati rice and other rice varieties. Three traits including length-to-width ratio of grain (L/W ratio), panicle length (PL), and amylose content (AC) showed significant (*P* < 0.05 and *P* < 0.01) differences between the *aromatic* and *indica*/*aus* groups. Comparative analysis of genome structure, based on genome sequence variation and GO analysis, revealed that the Basmati genome was derived mostly from the *aus* and *japonica* groups. Overall, whole-genome sequence data and genetic diversity information obtained in this study will serve as an important resource for molecular breeding and genetic analysis of Basmati varieties.

## Introduction

Rice (*Oryza sativa* L.) is an important cereal crop and represents the staple food of more than half of the global population (Wang and Li, 2005). *O. sativa* is classified into two distinct subspecies, *japonica* and *indica* (Kato, 1928), and into five groups including *indica*, *aus*, *aromatic*, temperate *japonica*, and tropical *japonica* (Garris et al., 2005). *O. sativa* was domesticated more than 10,000 years ago from Asian wild rice species, *O. rufipogon* and *O. nivara* (Kovach et al., 2007; Sang and Ge, 2007; Chen et al., 2019). Both *japonica* and *indica* rice have undergone significant phenotypic changes compared with *O. rufipogon* (proto-*japonica*) and *O. nivara* (proto-*indica*), respectively, and have expanded their geographical distribution during domestication (Fuller et al., 2010).

Basmati rice is considered a unique varietal group because of its aroma and superior grain quality (Ahuja et al., 1995; Siddiq et al., 2012). These unique varietal group occupies a special status among the consumers due to its unique quality traits such as extra-long slender grain, lengthwise excessive kernel elongation upon cooking, soft and fluffy texture after cooking, and aroma. Therefore, Basmati varieties are designated as the most highly produced and economically successful group (Civáň et al., 2019). The term Basmati is derived from two Sanskrit words, “*Vas*” meaning “*aroma*” and “*matup*” meaning “possessing.” The combination of the two Sanskrit words, “Vaasmati,” is pronounced as “Basmati” (Siddiq et al., 2012). Studies suggest that Basmati rice varieties represent the *aromatic* group from *indica* and *japonica* subspecies (Glaszmann, 1987; Garris et al., 2005).

From the decades, less attention has given at the origin of Basmati group. This is mainly due to the conflicting phylogenetic relationships were observed among Basmati and other rice groups (Choi et al., 2017). Furthermore, genome-wide polymorphism analysis in Asian cultivated rice showed that Basmati rice varieties share a close phylogenetic relationship with *japonica* varieties (Huang et al., 2012; Wang et al., 2018). Recent findings of Choi et al. (2018) and Civáň et al. (2019) providing more evidence that Basmati genome was genetically close to *japonica* and *aus* rice. However, these studies were carried out using single Basmati genome, which has limited information on Basmati genome variation. Although some progresses have been made in understanding of origin of Basmati genome, further study is needed to identify the Basmati-specific genome features and genome variation by assembling the traditional Basmati varieties compared with *japonica*, *indica*, and *aus* groups. Next-generation sequencing (NGS) technologies are important for genomic analysis and molecular breeding (Chen et al., 2014), and enable the identification of functional genomic variation, and unique SNPs, and insertion-deletion polymorphisms (InDels) across the genome, which offer an exciting opportunity to genetic diversity studies in the crop plants (Jimenez et al., 2013; Serba et al., 2019).

In Basmati rice, molecular mapping and cloning of the *fgr* gene, which encodes betaine aldehyde dehydrogenase homologue 2 (*Badh2*), revealed an 8-bp deletion and three single nucleotide polymorphisms (SNPs) in the 7th exon, resulting in the fragrant trait (Bradbury et al., 2005). Haplotype analysis of the *Badh2* gene showed that the 8-bp deletion in the majority of fragrant Basmati varieties causes a loss-of-function mutation, which enhances the biosynthesis of 2-acetyl-1-pyroline (2-AP); this haplotype is identical to the ancestral *japonica* haplotypes, suggesting that introgression between *japonica* accessions and Basmati varieties is responsible for the fragrant trait in Basmati rice (Kovach et al., 2009). A recent study by Daygon et al. (2017) reported that four other amine heterocycles: 6-methyl, 5-oxo-2,3,4,5-tetrahydropyridine (6M5OTP), 2-acetylpyrrole, pyrrole, and 1-pyrroline, that correlate strongly with the production of 2AP, and are present in consistent proportions in a collection of recombinant inbred lines derived from Basmati-type rice, and these compounds were also co-localized with a single QTL that harbors the *fgr* gene. Although genetic basis of fragrant trait in Basmati rice seems to be complicated, most researchers proposed that grain aroma in Basmati rice is controlled by a single recessive gene (*Badh2*) (Bradbury et al., 2005; Kovach et al., 2009). However, some researchers also think that fragrant trait in Basmati rice is controlled by major and minor-effective genes (Daygon et al., 2017), and by several QTLs (Amarawathi et al., 2008; Pachauri et al., 2014; Vemireddy et al., 2015). Overall, the molecular genetic mechanism of fragrant trait is not clearly understood, more studies is needed on the functional allelic variation of aroma gene and number of genes controlling the grain aroma in Basmati rice.

In this study, we analyzed the differences between Basmati rice genome *vs. indica*, *japonica*, and *aus* rice genomes through whole-genome sequencing and marker analysis. The main objective is to identify the genomic features and genetic variation in Basmati rice that can be utilized for genetic studies and marker development for breeding. We also identified unique SNPs and Indel marker sets, and evaluated the key agronomic and grain quality traits of Basmati rice with other rice groups for varietal improvement.

## Materials and Methods

### Plant Materials

**Figure 1:**
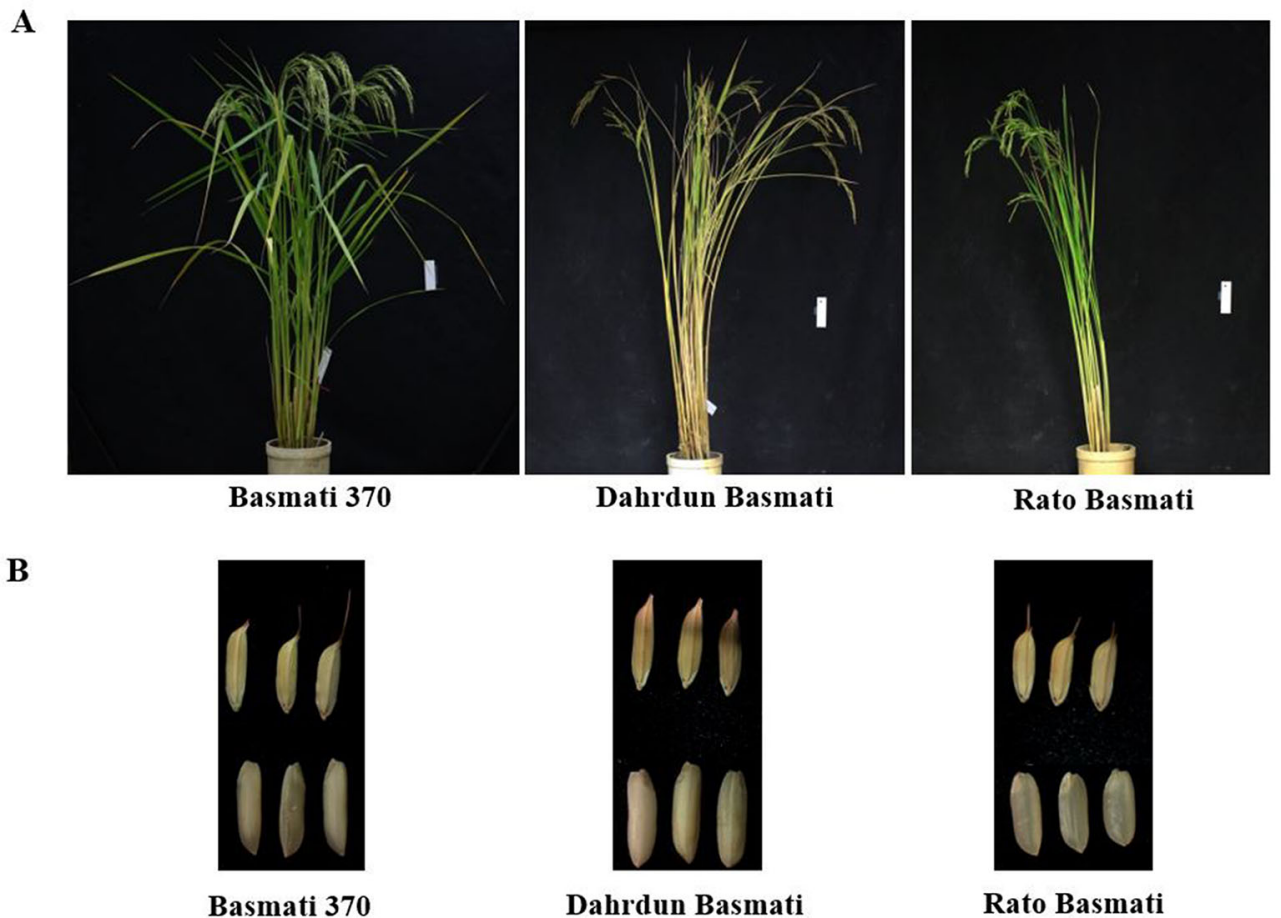
Phenotypic comparison of Basmati 370, Dahrdun Basmati, and Rato Basmati. **(A)** Plant phenotype. **(B)** Spikelet and mature grain.

A total of 60 rice varieties belonging to *indica*, *aus*, *aromatic*, temperate *japonica*, and tropical *japonica* groups were used in this study (**Table 1**). Among the 60 rice varieties, seeds of 30 traditional Basmati varieties [International Rice GenBank Collection (IRGC) designated] were obtained from the International Rice Research Institute (IRRI), while the other 30 rice varieties were from the Crop Molecular Breeding Lab, Seoul National University. Among the 30 traditional Basmati varieties, Basmati 370, Rato Basmati, and Dahrdun Basmati were selected for whole-genome resequencing, based on their geo-location (**Figure 1**). Seeds from each accession were surface sterilized and sown in pots containing wet soil. The pots were placed in an experimental greenhouse for 30 days. Then, 30-day-old seedlings were transplanted in an experimental field at Seoul National University.

**Table 1:** List of rice varieties used in this study.

No.	Varieties	Origin	Accession no. *^a^*	Subgroup*^b^*	*Badh2* allele*^c^*
1	Nipponbare	Japan	981704	Temperate *japonica*	WT
2	Koshihikari	Japan	981581	Temperate *japonica*	WT
3	Yukara	Japan	981584	Temperate *japonica*	WT
4	Ilpumbyeo	South Korea	981585	Temperate *japonica*	WT
5	Jinheungbyeo	South Korea	981576	Temperate *japonica*	WT
6	Dongjinbyeo	South Korea	981626	Temperate *japonica*	WT
7	Hopumbyeo	South Korea	980403	Temperate *japonica*	WT
8	Tong 88-7	South Korea	980609	Temperate *japonica*	WT
9	MS 11	Philippines	981589	Temperate *japonica*	WT
10	Samnambyeo	South Korea	981579	Tropical *japonica*	WT
11	Malagkit Sinaguing	Philippines	961354	*Admixture*	WT
12	B581A6	Philippines	921648	Tropical *japonica*	WT
13	CP-SLO	USA	970083	Tropical *japonica*	WT
14	Azucena	Philippines	971155	Tropical *japonica*	WT
15	Reket Abang	Indonesia	260004	Tropical *japonica*	WT
16	Dawn	USA	981564	Tropical *japonica*	WT
17	Milyang 23	South Korea	981599	*Indica*	WT
18	Dasanbyeo	South Korea	981598	*Indica*	WT
19	Taichung Native 1	Taiwan	981570	*Indica*	WT
20	IR 64	Philippines	981566	*Indica*	WT
21	IR 72	Philippines	18053	*Indica*	WT
22	Chinsurah Boro 2	Bangladesh	851453	*Aus*	WT
23	Dular	India	980384	*Aus*	WT
24	Bina Dhan 10	Bangladesh	961192	*Indica*	WT
25	IR 24	Philippines	18049	*Indica*	WT
26	IR 8	Philippines	981596	*Indica*	WT
27	Minghui 63	China	981601	*Indica*	WT
28	N 22	India	970030	*Aus*	WT
29	Swarna	India	961181	*Indica*	WT
30	Basmati Dhan	Nepal	IRGC 23814	*Aromatic*	*badh2.1*
31	Dheradun Basmati	Nepal	IRGC 23861	*Aus*	WT
32	Basmati Nahan 381	Pakistan	IRGC 27786	*Aromatic*	*badh2.1*
33	Basmati Sufaid 100	Pakistan	IRGC 27791	*Aromatic*	*badh2.1*
34	Basmati 140	Pakistan	IRGC 27813	*Aus*	WT
35	Basmati 370	Pakistan	IRGC 27820	*Aromatic*	*badh2.1*
36	Basmati 372	Pakistan	IRGC 27823	*Aromatic*	*badh2.1*
37	Basmati 377	Pakistan	IRGC 27829	*Aromatic*	*badh2.1*
38	Deraduni Basmati 321	Pakistan	IRGC 27907	*Aromatic*	*badh2.1*
39	Kamoh Basmati 392	Pakistan	IRGC 28000	*Aromatic*	*badh2.1*
40	Sathi Basmati	Pakistan	IRGC 28230	*Aromatic*	*badh2.1*
41	Basmati Sal	India	IRGC 52411	*Aus*	WT
42	Basmati Kunar	Afghanistan	IRGC 58272	*Aromatic*	*badh2.1*
43	Basmati Kunduz	Afghanistan	IRGC 58273	*Indica*	WT
44	Basmati Anpjhutte	Nepal	IRGC 58879	*Aromatic*	*badh2.1*
45	Basmati Gola	Nepal	IRGC 58880	*Aromatic*	*badh2.1*
46	Basmati Lamo	Nepal	IRGC 58881	*Aromatic*	*badh2.1*
47	Basmati Masino	Nepal	IRGC 58883	*Indica*	WT
48	Basmati Nokhi	Nepal	IRGC 58884	*Indica*	WT
49	Basmati Pahade	Nepal	IRGC 58885	*Aromatic*	*badh2.1*
50	Basmati Red	Nepal	IRGC 58886	*Aromatic*	*badh2.1*
51	Basmati White	Nepal	IRGC 58887	*Aromatic*	*badh2.1*
52	Basmati Uzarka	Nepal	IRGC 58888	*Aromatic*	*badh2.1*
53	Kalo Basmati	Nepal	IRGC 59054	*Aromatic*	*badh2.1*
54	Rato Basmati	Nepal	IRGC 59205	*Aromatic*	*badh2.1*
55	Basmati Mwea	Kenya	IRGC 61183	*Aromatic*	*badh2.1*
56	Dahrdun Basmati	India	IRGC 67705	*Indica*	WT
57	Basmatiya	India	IRGC 67734	*Aus*	WT
58	Pakistani Basmati	India	IRGC 67746	*Aromatic*	*badh2.1*
59	Karnal Basmati	Pakistan	IRGC 76362	*Aromatic*	*badh2.1*
60	Kasalath	India	980341	*Indica*	WT

a IRGC, International Rice GenBank Collection.

b Subgroup was determined based on 190 SNP markers.

c Badh2 genotype was determined based on the *fgr*-specific InDel marker developed by Sakthivel et al. (2009) and WT indicates wild type allele.

### Genome Sequencing

**Supplementary Figure 1** provides an overview of the work plan used in this study. To perform whole-genome resequencing, shotgun DNA libraries were prepared from high molecular weight genomic DNA of three traditional Basmati varieties using the NEXTflex™ Rapid DNA-Seq kit (Bioo Scientific Corporation, Austin, TX, USA). Then, the libraries were used for cluster generation and sequenced for 250 cycles on the Illumina HiSeq2500 platform (Illumina, San Diego, CA, USA), according to the manufacturer’s instructions, at the National Instrumentation Center for Environmental Management (NICEM) of Seoul National University.

### Mapping and SNP Discovery

Raw sequence reads were subjected to quality trimming using FastQC v0.11.3 (http://www.bioinformatics.babraham.ac.uk/projects/fastqc/), and reads with a Phred quality (Q) score <20 were discarded. Adapter trimming was carried out by using Trimmomatic (http://www.usadellab.org/cms/?page=trimmomatic). The clean reads were mapped to the reference genomes of the temperate *japonica* cultivar Nipponbare (Os-Nipponbare-Reference-IRGSP-1.0; Kawahara et al., 2013), *indica* cultivar Zhenshan 97 (Os-Zhenshan 97-Reference; Zhang et al., 2016), and *aus* cultivar Kasalath (Os-Kasalath-Reference; Sakai et al., 2014) using the Burrows–Wheeler Aligner (BWA) program (Li and Durbin, 2010). The alignment results were merged and converted into binary alignment map (BAM) files (Barnett et al., 2011). The BAM files were used to calculate the sequencing depth and to identify SNPs and InDels using the GATK program, with default parameters (McKenna et al., 2010).

### Genomic Analysis

The genic and intergenic distribution of SNPs and InDels was determined relative to Nipponbare, Zhenshan 97, and Kasalath reference genomes. The distribution of genic SNPs and InDels common to the three Basmati genomes were presented using Circos (Krzywinski et al., 2009).

*In silico* analysis was performed to identify Basmati-specific SNPs and InDels using resequencing data of 54 diverse rice varieties in the Crop Molecular Breeding Lab, Seoul National University database (unpublished data) and Rice Variation Map v2.0 public database (http://ricevarmap.ncpgr.cn/v2/). InDel in nine traditional Basmati varieties and 11 *indica*, *aus*, and *japonica* check varieties were verified by gel electrophoresis, based on *in silico* analysis, using primers designed with Primer-3 (http://bioinfo.ut.ee/primer3-0.4.0/).

### GO Analysis

The annotated Nipponbare, Zhenshan 97, and Kasalath reference genes were classified based on the pattern of common SNPs in the three Basmati genomes. Functional annotation of genes was investigated with “*Oryza sativa*” as the background species. GO analysis was performed using the BLAST2GO software (www.blast2go.com) (Conesa et al., 2005). Whole-genome orthologous gene comparison, annotation, and clustering were performed using the Orthovenn program (Wang et al., 2015).

### DNA Extraction and Genome-Wide SNP Marker Analysis

Genomic DNA was isolated from the leaf tissues of plants at the 3–4 leaf stage using the modified cetyltrimethylammonium bromide (CTAB) method (McCouch et al., 1988). DNA concentration and quality were determined using the NanoDrop spectrophotometer (Thermo Scientific, Wilmington, NC, USA).

On the basis of differences in DNA sequences between *indica* and *japonica* genomes, 190 subspecies-specific SNP markers, representing all 12 rice chromosomes, were developed in the Crop Molecular Breeding Lab, Seoul National University (unpublished data). SNP genotyping was conducted on Fluidigm 96.96 Dynamic Arrays using the BioMark HD System (Fluidigm Corp, San Francisco, CA), according to the manufacturer’s instructions, and genotypes were determined using the Fluidigm SNP Genotyping Analysis software.

### Phylogenetic and Population Structure Analyses

Phylogenetic analysis was performed using PowerMarker v3.25 (Liu and Muse, 2005). Cavalli-Sforza and Edwards (1967) genetic distance was used to construct an unweighted pair group method with an arithmetic average (UPGMA) dendrogram, which was visualized in Molecular Evolutionary Genetics Analysis 7 (MEGA7) (Kumar et al., 2016). The population structure of 60 rice varieties was determined using a model-based approach available in the STRUCTURE 2.3.4 software (Falush et al., 2003). The number of genetically distinct populations (*K*) was adjusted from 1 to 10, and the model was repeated three times for each *K*. The burn in-period was adjusted with 100,000 iterations, followed by 100,000 Markov Chain Monte Carlo (MCMC) per run. The best *K* value was determined based on delta *K* (Δ*K*) using the Evanno method in the web-based python program, STRUCTURE HARVESTER (Earl and Vonholdt, 2012).

### *Badh2* Marker Analysis

All 60 rice varieties were classified as *badh2.1* and wild *Badh2* allele harboring genotypes by PCR-based genotyping of the *Badh2* InDel marker using the forward primer 5′-TGTTTTCTGTTAGGTTGCATT-3′ and reverse primer 5′-ATCCACAGAAATTTGGAAAC-3′ (Sakthivel et al., 2009). PCR was conducted using the following conditions: initial denaturation at 94°C for 2 min, followed by 35 cycles of denaturation at 95°C for 20 s, annealing at 54°C for 30 s, and extension at 72°C for 30 s, and a final extension at 72°C for 1 min. The amplified products were separated by electrophoresis on 3.5% agarose gel.

### Agronomic and Grain Quality Trait Analyses

Passport data on 13 agronomic and grain quality traits of 30 traditional Basmati varieties, including days to heading (DH), leaf width (LW), days to maturity (DM), culm length (CL), culm number (CN), culm diameter (CD), grain length (GL), grain width (GW), length-to-width ratio of grain (L/W ratio), 1,000 grain weight (KGW), panicle length (PL), spikelet fertility count (SFC), and amylose content (AC), were obtained from Genesys (https://www.genesys-pgr.org). Cluster analysis and Student’s *t*-test were performed using SPSS 16.0 (https://www.ibm.com/analytics/spss-statistics-software).

## Results

### Basmati Genome Sequencing

High-throughput sequencing of three traditional Basmati varieties was performed to facilitate downstream analysis. A total of 43,024,210 reads were generated from Basmati 370; 43,263,296 reads from Dahrdun Basmati; and 44,099,730 reads from Rato Basmati, each corresponding to more than 10 GB read length, and more than 90% of these reads were clean reads (**Table 2**). The clean reads were mapped to the reference genomes of Nipponbare (*japonica* rice), Zhenshan 97 (*indica* rice), and Kasalath (*aus* rice). The mapping results indicated that all genomes were sequenced at a depth ranging from 26.02X to 30.75X, with more than 90% coverage.

**Table 2:** Data generated from whole-genome resequencing of three Basmati varieties.

Varieties	Raw reads		Clean reads		Coverage (%)
	Read number	Read length (bp)	Read number	Read length (bp)	
Basmati 370	43,568,684	10,935,739,684	43,024,210	10,117,316,665	92.52
Dahrdun Basmati	43,936,332	11,028,019,332	43,263,296	9,971,121,538	90.42
Rato Basmati	44,616,386	11,198,712,886	44,099,730	10,236,348,518	91.41

The number of SNPs in each Basmati variety were determined relative to each reference genome. Compared with Nipponbare, we identified 1,544,399 SNPs in Basmati 370; 2,105,019 SNPs in Dahrdun Basmati; and 1,229,155 SNPs in Rato Basmati. Similarly, comparison with the Kasalath reference genome revealed 1,453,259 SNPs in Basmati 370; 1,336,541 in Dahrdun Basmati; and 1,627,481 SNPs in Rato Basmati, whereas comparison with the Zhenshan 97 reference genome revealed 1,409,129 SNPs in Basmati 370; 793,929 SNPs in Dahrdun Basmati; and 1,659,254 SNPs in Rato Basmati. Thus, Dahrdun Basmati showed the highest number of SNPs compared with Nipponbare and the lowest number of SNPs compared with Zhenshan 97 (**Table 3**).

**Table 3:** Mapping and SNP summary of three traditional Basmati varieties.

Reference varieties	Basmati varieties	Mapping information					SNP data						
		Raw reads	Mapped reads	Unmapped reads	Average depth	Coverage (%)	Non-synonymous	Synonymous	Intron	5′UTR	3′UTR	Intergenic	Total
Nipponbare	Basmati 370	43,024,210	40,389,926	578,354	27.06	91.04	35,381	30,767	25,440	15,887	31,829	1,405,095	1,544,399
	Dahrdun Basmati	43,263,296	40,402,732	498,038	26.52	90.07	45,978	40,181	31,729	21,029	43,401	1,922,701	2,105,019
	Rato Basmati	44,099,730	42,127,262	405,096	27.57	92.07	28,484	24,751	19,335	12,643	25,573	1,118,369	1,229,155
Kasalath	Basmati 370	43,024,210	38,696,238	1,149,044	30.11	96.33	14,004	10,905	31,589	1,876	3,935	1,390,950	1,453,259
	Dahrdun Basmati	43,263,296	39,076,602	988,102	29.08	96.63	13,409	10,759	30,844	1,875	4,099	1,275,555	1,336,541
	Rato Basmati	44,099,730	40,369,808	969,672	30.75	96.08	14,796	11,394	33,791	1,929	4,225	1,561,346	1,627,481
Zhenshan 97	Basmati 370	43,024,210	38,936,652	1,673,302	27.06	91.79	95,093	62,744	89,502	23,344	44,958	1,093,488	1,409,129
	Dahrdun Basmati	43,263,296	39,734,380	1,442,176	26.02	94.90	54,599	36,577	50,150	13,471	25,394	613,738	793,929
	Rato Basmati	44,099,730	40,356,836	1,510,382	27.80	90.33	111,918	73,592	103,813	28,359	53,212	1,288,360	1,659,254

In comparison with the Nipponbare reference genome, relatively high numbers of SNPs were detected on chromosomes 1, 3, 6, and 11 in Basmati genomes, while the lowest numbers of SNPs were detected on chromosomes 9 and 5. Compared with Kasalath, Basmati varieties showed a high proportion of SNPs on chromosomes 1, 2, 3, 6, and 7, and the lowest numbers of SNPs on chromosome 10. Compared with Zhenshan 97, we found a high proportion SNPs on chromosomes 1, 2, 6, and 7 in Basmati varieties and lower SNP numbers on chromosomes 5 and 9. The distribution of SNPs on all 12 chromosomes of the three Basmati varieties in comparison with all three reference genomes is summarized in **Supplementary Table 1**.

Furthermore, we also determined the number of SNPs and InDels in each Basmati variety against the three reference genomes. Accordingly, in **Supplementary Table 2**. In comparison with Nipponbare, InDels were abundant on chromosomes 3 and 6 in Basmati varieties, while the number of SNPs was the highest on chromosome 1. In comparison with Kasalath and Zhenshan 97 reference genomes, chromosomes 1, 2, and 3 of Basmati varieties contained a high proportion of InDels, while chromosomes 1, 2, and 6 showed the highest number of substitutions.

### Distribution of Common SNPs and InDels in Genic Regions

**Figure 2:**
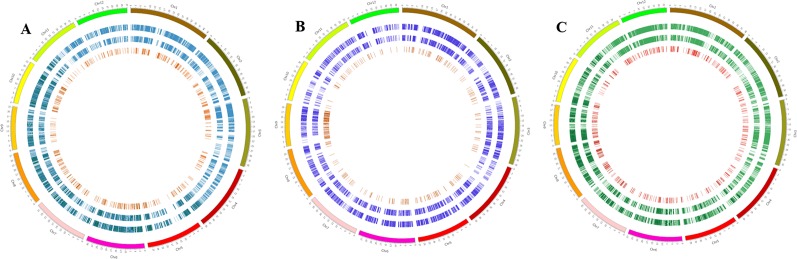
Circos plots showing the distribution pattern of SNPs and InDels in the genic regions of three Basmati varieties. **(A**–**C)** Distribution of SNPs and InDels in Basmati varieties in comparison with Nipponbare **(A)**, Kasalath **(B)**, and Zhenshan 97 **(C)** reference genomes. The outermost circle represents 12 chromosomes of the rice genome. The second circle from the outside represents common SNPs. The third circle from the outside represents functional SNPs. The innermost circle with red bars shows the distribution of InDels ranging in size from 5 to 30 bp.

Common SNPs in genic regions, functional SNPs [non-synonymous SNPs and SNPs in untranslated regions (UTRs)], and InDels (5–30 bp) in genic regions were identified by comparing all three Basmati genomes with all three reference genomes. The total number of common SNPs identified in Basmati varieties were 52,204 compared with Nipponbare; 19,207 compared with Kasalath; and 73,219 compared with Zhenshan 97. The extracted common SNPs were plotted within the Nipponbare (**Figure 2A**), Kasalath (**Figure 2B**), and Zhenshan 97 (**Figure 2C**) reference genomes.

In addition, *in silico* analysis using resequencing data of 54 varieties revealed 20 novel unique SNPs in genic regions of the Basmati genomes. These unique SNPs were also confirmed using the public rice database (http://ricevarmap.ncpgr.cn/v2/). Additionally, we identified 11 unique InDels in the Basmati genomes. The unique SNPs and InDels, and the functions of genes containing these polymorphisms, are listed in **Supplementary Table 3**. PCR amplification of 289 bp fragments using gene-specific primers (forward primer, 5′-CTGTTTATACGTAGTACGGGTTG-3′; reverse primer, 5′-TGTTTGTAGGGGGATGCAAT-3′), which confirmed that the 25 bp insertion in the intron of the gene involved in seed development regulation (*Os10g0139300*; IRGSP-1.0; position: 2,425,049 bp) was only specific to the Basmati and *aus* groups, and could be discriminated among 20 rice varieties (**Supplementary Figure 2**). We further examined the spatiotemporal expression pattern of *Os10g0139300* in the RiceXpro database (Sato et al., 2011); this gene showed high expression in the embryo and endosperm after flowering, indicating a possible role in seed development during ripening.

### GO Analysis of Basmati Varieties

**Figure 3:**
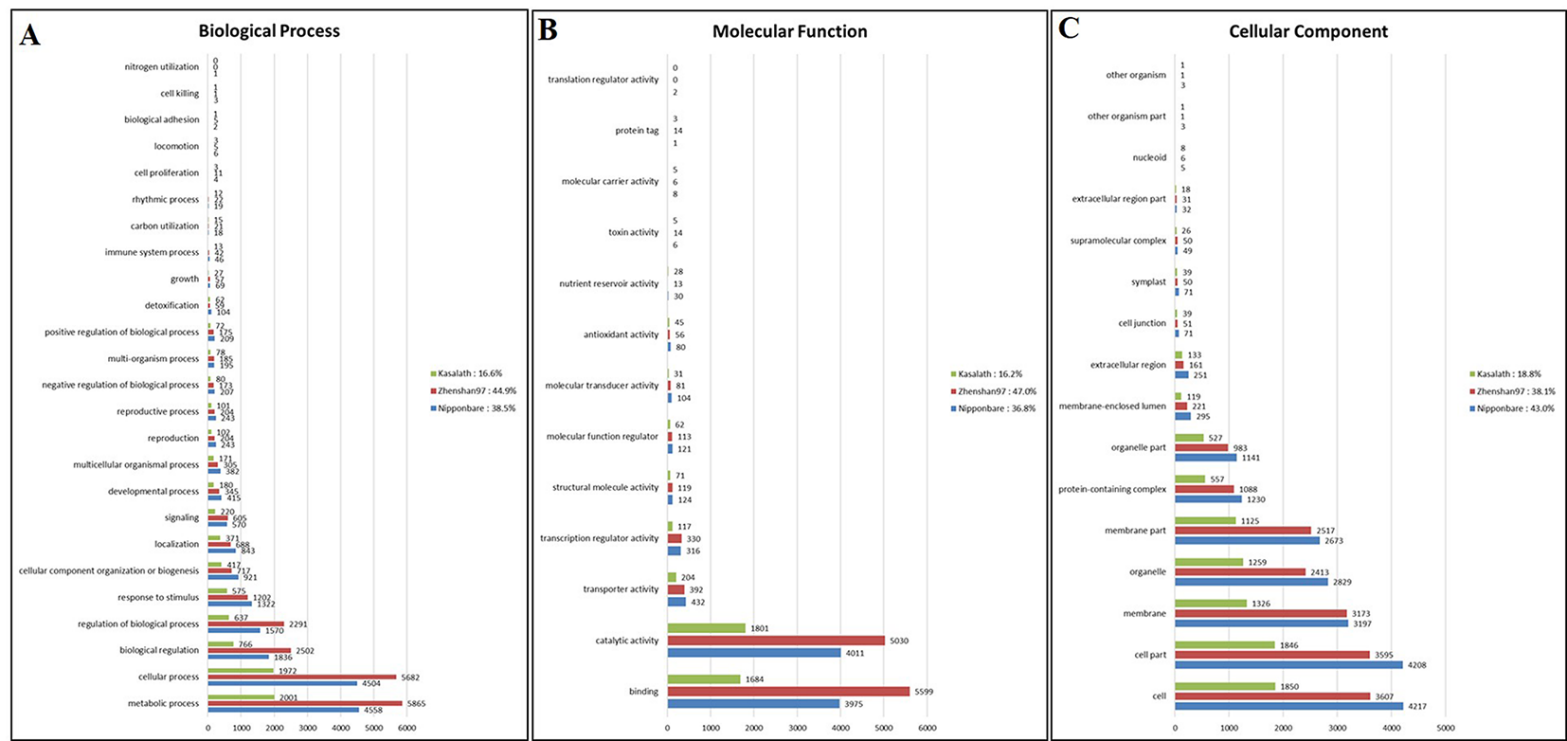
Gene ontology (GO) analysis of Basmati genomes in comparison with Zhenshan 97, Kasalath, and Nipponbare genomes. **(A**–**C)** GO categories including biological process **(A)**, molecular function **(B)**, and cellular component **(C)** are shown.

We investigated the functions of genes containing common SNPs and InDels among the three Basmati genomes *via* GO analysis. Genes were assigned to three categories, namely, biological process (BP), molecular function (MF), and cellular component (CC). The major GO associations were found in metabolic process, cellular process, biological regulation for BP terms (**Figure 3A**). For the MF terms, binding and catalytic activity (**Figure 3B**). Whereas, cell, cell part, and membrane were associated with CC terms (**Figure 3C**).

**Figure 4:**
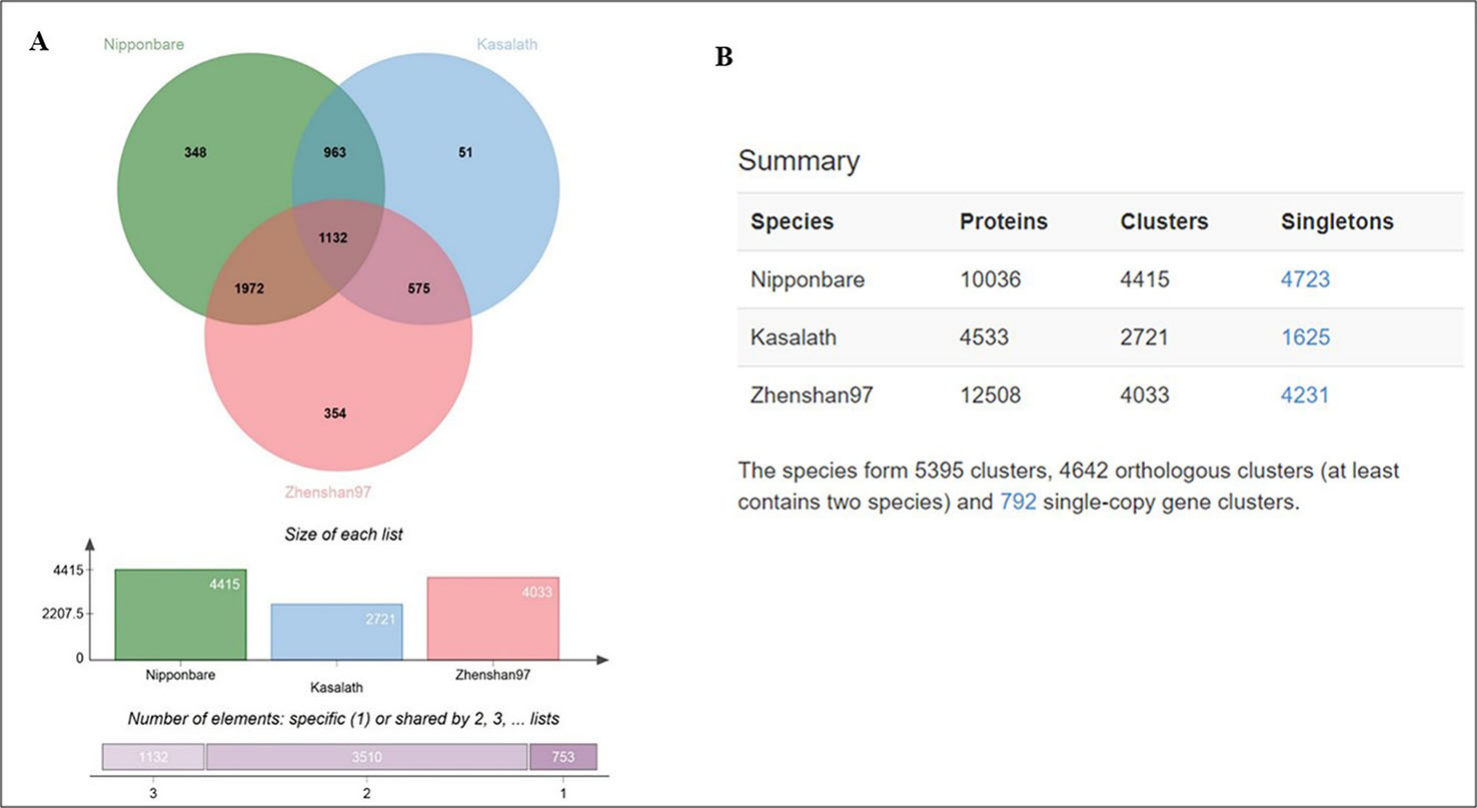
Ortho Venn diagram. **(A)** Venn diagram showing the distribution of shared gene families among Nipponbare, Kasalath, and Zhenshan 97. Specific gene clusters are indicated within the three reference genomes. **(B)** Counts of clusters in each genome.

Furthermore, we analyzed genome-wide orthologous clusters of genes from Basmati varieties using common SNPs by comparison with Nipponbare, Zhenshan 97, and Kasalath reference genomes. The analysis revealed 5,395 orthologous clusters based on protein sequences of the three reference genomes (**Figure 4A**). The Venn diagram showed that 1,132 gene clusters were shared by all three reference genomes, suggesting their conservation in the lineage after speciation (**Figure 4B**). Additionally, 348, 354, and 51 clusters specific to Nipponbare, Zhenshan 97, and Kasalath reference genomes, respectively, were identified. Additionally, cluster analysis of the mutated genes in the three Basmati varieties revealed 4,415 clusters in comparison with Nipponbare; 2,721 clusters in comparison with Kasalath; and 4,033 clusters in comparison with Zhenshan 97 reference genomes. The presence of 2,721 clusters in comparison with Kasalath suggests that Basmati varieties show less genetic variation compared with the *aus* group.

In phylogenetic studies, the identification of single-copy orthologs is critical in any species (Creevey et al., 2011). Orthologous cluster analysis revealed 792 clusters representing single-copy genes, which were shared by all three reference genomes, suggesting that the single-copy status of genes was maintained during evolution after species divergence.

**Figure 5:**
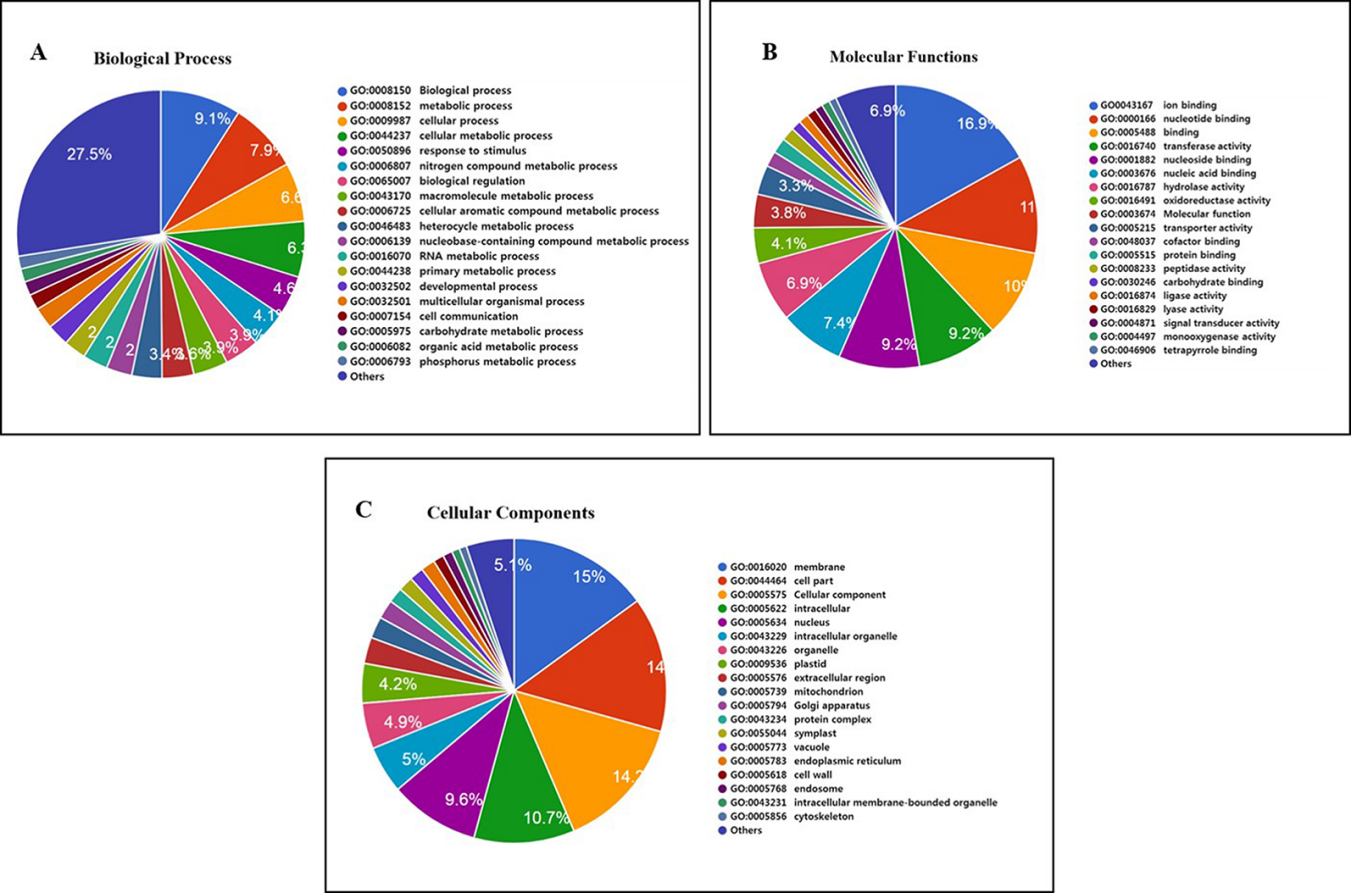
Functional annotation of 1,132 mutated gene cluster of Basmati genome shared by Nipponbare, Kasalath, and Zhenshan 97 reference genomes. **(A)** Biological process. **(B)** Molecular function. **(C)** Cellular component.

Furthermore, 1,132 gene clusters shared by Nipponbare, Kasalath, and Zhenshan 97 reference genomes harbored unique SNPs from all three Basmati varieties, and functional annotation of the genes harboring these unique SNPs showed that the majority of these genes were involved in biological regulation, metabolic process, and cellular process (**Figure 5A**); binding and catalytic activity (**Figure 5B**); and membrane, cell parts, and cellular component (**Figure 5C**). We also detected mutated gene clusters associated with the metabolic process involved in the cellular aromatic compound (**Figure 5A**). Further, a total of 35 genes including *Badh2* gene were found to be involved in aromatic compound biosynthesis based on biological process and molecular functional annotation. While, genomic regions from three Basmati varieties compared to Nipponbare reference genome showed functional variation across the 35 genes involved in aromatic compound biosynthesis (data not shown). Whereas, *in sillico* analysis of 35 genes using Rice Variation Map v2.0 revealed that only nine genes including *Badh2* gene having alternative alleles in 96 varieties of aromatic group with more than 80% of frequency.

### SNP Genotyping and Genetic Relationship

To determine the genetic relationship of 30 traditional Basmati varieties, including three resequenced Basmati varieties, with rice varieties belonging to other groups, a total of 60 varieties were genotyped with two sets of 96-plex *indica/japonica* SNPs. Two of these SNP markers were excluded from the analysis because of their low quality. The number of SNP markers, average physical interval between SNPs per chromosome, and coverage percentage are summarized in **Supplementary Table 4**.

All 190 SNP markers were biallelic between *indica* and *japonica* varieties, and the average allele number was 2.12. In addition, the average value of major allele frequency (MAF) was 0.681, and almost all SNPs showed no heterozygosity (average heterozygosity = 0.020). Consistent with these data, the average polymorphic information content (PIC) was 0.33 (**Supplementary Table 5**).

**Figure 6:**
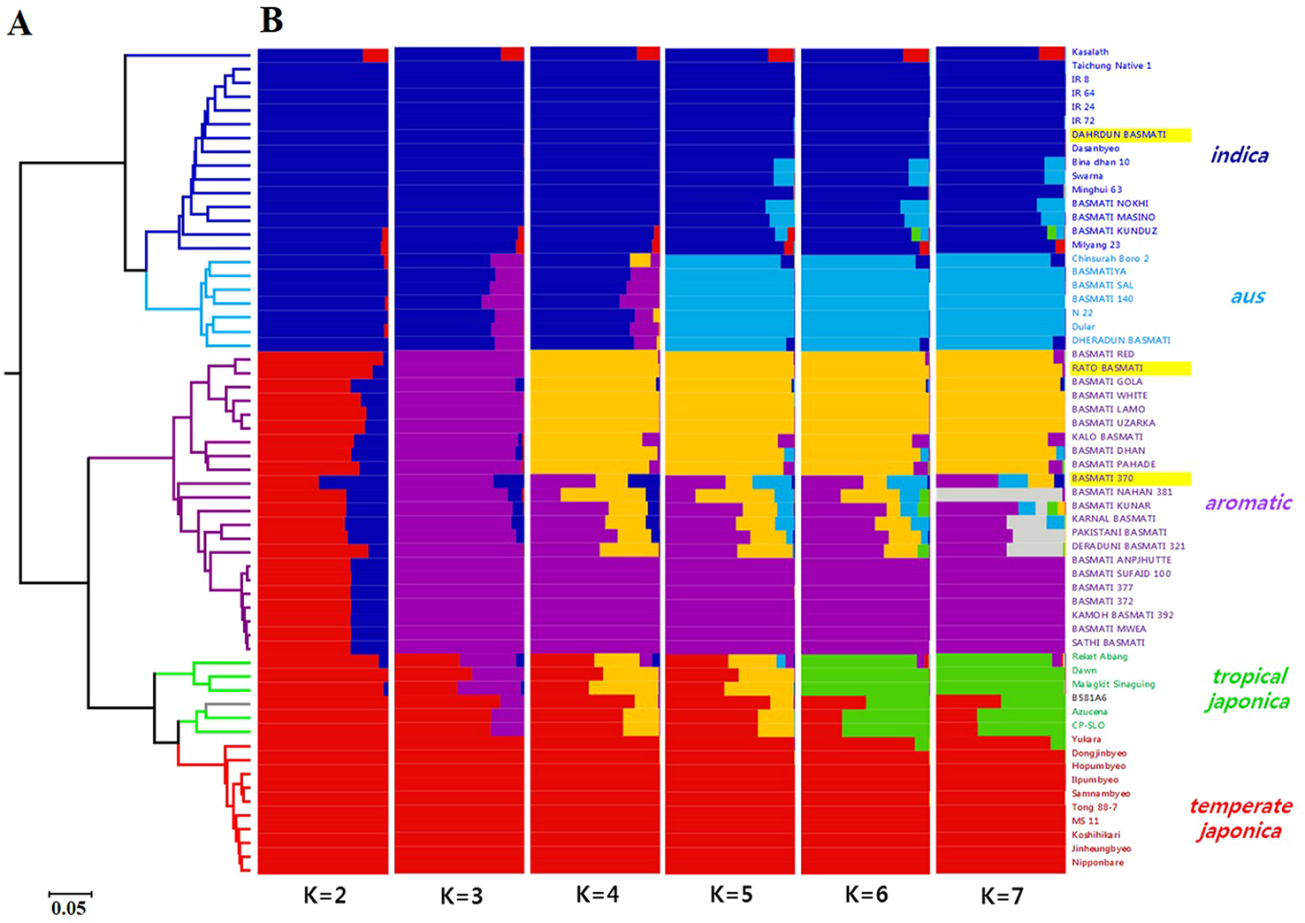
Genetic diversity analysis of 60 rice varieties using 190 SNPs. **(A)** UPGMA dendrogram. The branches are colored according to the subpopulation assessment in **(B)** based on *K* = 6, except for the *aromatic* group, which is based on *K* = 3. Gray branches indicate admixture. **(B)** Population structure analysis using the STRUCTURE software for *K* values ranging from 2 to 7. Three varieties used for genomic analysis are highlighted in yellow.

The UPGMA dendrogram based on Cavalli-Sforza and Edwards (1967) genetic distance (**Figure 6A**) classified all 60 varieties into two subspecies, *indica* and *japonica*. Additionally, the *japonica* group showed two distinct subgroups, *aromatic* and *japonica*. The 30 Basmati varieties were divided into two groups, *indica* (comprising Dahrdun Basmati) and *japonica* (comprising Rato Basmati and Basmati 370). To identify the population structure of all 60 rice varieties, STRUCTURE analysis was carried out. The value of delta *K* was maximum at *K* = 2 (**Supplementary** **Figure 3**). At *K* = 2, 60 varieties were classified into *indica* and *japonica*, as expected based on marker characteristics; however, more than half of the varieties in the *japonica* group showed admixture with *indica* ancestry. At *K* = 3, the *aromatic* group along with Rato Basmati and Basmati 370 grouped at the *japonica* group, and at *K* = 4, the *aromatic* group was divided into two clear subgroups and one admixed group. All nine varieties, including Rato Basmati, in the upper yellow subgroup within the *aromatic* group (**Figure 6B**), were from Nepal. At *K* = 6, five subgroups were evident among the 60 varieties including *indica*, *aus*, *aromatic*, tropical *japonica*, and temperate *japonica*, except one variety, which showed less than 65% of estimated ancestry derived from any single subgroup (**Figure 6B**). The results of phylogenetic and population structure analyses were consistent. Among the 30 traditional Basmati varieties, four varieties, including Dahrdun Basmati, were classified into the *indica* group; four into the *aus* group; and 22, including Rato Basmati and Basmati 370, into the *aromatic* group.

### *Badh2* Marker Analysis

Among 60 rice varieties, 30 traditional Basmati varieties were further investigated on the basis of the 8 bp deletion in the *Badh2* gene to classified into *badh2.1* and wild *Badh2* allele harboring genotypes. PCR-based genotyping of the *Badh2* InDel marker divided the traditional Basmati varieties into two groups: *badh2.1* (22 varieties; 95 bp PCR product) and wild *Badh2* allele carrying genotypes (8 varieties; 103 bp PCR product) (**Supplementary Figure 4**, **Table 1**). The remaining 30 non-Basmati rice varieties were classified in the wild *Badh2* allele group (**Table 1**).

### Agronomic and Grain Quality Trait Analysis

The mean performance of 13 agronomic and grain quality traits of 30 traditional Basmati varieties is presented in **Supplementary Table 6**. The coefficient of variation of CN was the highest (25.49), followed by that of SFC (24.87). Comparison of the mean performance between *aromatic* and *indica/aus* groups revealed significant differences in only L/W ratio, PL, and AC; the *aromatic* group showed significantly longer panicles, longer and slender grains, and lower AC than the *indica/aus* group (**Supplementary Table 7**).

Next, hierarchical cluster analysis was performed to elucidate the relationship among the 30 traditional Basmati varieties. These varieties were divided into two major clusters (I and II), and each cluster was further divided into three subclusters (**Supplementary Figure 5**, **Supplementary Table 6**). Cluster I contained 20 moderate duration varieties from diverse geographical regions with superior grain quality. Cluster II consisted of ten late duration varieties, mostly from Nepal, with poor grain quality; thus cluster II showed less genetic diversity than cluster I.

## Discussion

Basmati rice varieties, considered a unique varietal group, have been generally classified into the *aromatic* group (Glaszmann, 1987; Garris et al., 2005; Civáň et al., 2015). Recent findings suggest that Basmati rice was derived mostly from *aus* and *japonica* varietal groups (Civáň et al., 2015). Recently, the genome assembly of Basmati rice was performed using “Basmati Surkh 89-15,” an improved cultivar from Pakistan (Zhao et al., 2018). However, a higher level of introgression from other rice populations in improved varieties of Basmati makes it difficult to define the genome structure. A latest preprint of phylogenomic analysis involving “Basmati 334” proposed admixture events between Basmati rice, *aus*, and *O. rufipogon*; this study concluded that Basmati rice has a hybrid origin and is closely related to both *japonica* and *aus* rice (Choi et al., 2018). However, phylogenomic analysis using a single genome cannot provide detailed information about the Basmati genome structure, when referring to the entire Basmati group, irrespective of the *Badh2* allele type. Therefore, defining Basmati-specific genome features is important to understand the domestication of Asian rice.

In this study, we performed whole-genome resequencing and analysis of three traditional Basmati varieties. The identification of genome-wide nucleotide polymorphisms, including SNPs and InDels, using NGS has gained importance in the rice genome (Markkandan et al., 2018) and has enabled researchers to identify genome-specific features in rice varieties. Therefore, we performed NGS data analysis of Basmati 370, Dahrdun Basmati, and Rato Basmati to characterize the Basmati genome in detail. We found that millions of SNPs in all Basmati varieties in comparison with Nipponbare, Kasalath, and Zhenshan 97 reference genomes (**Table 3**), thus providing an opportunity to identify Basmati-specific features. Additionally, the genome-wide common SNPs and InDels identified in this study would serve as a useful resource for the development of SNP and InDel markers for the Basmati genome, specific to *japonica*, *aus*, and *indica* varietal groups (**Figures 2A–C**). Similarly, *in silico* analysis of the three Basmati rice genomes along with 54 rice varieties revealed high-quality Basmati-specific features.

Basmati rice varieties showed less genomic variation compared with the *aus* group and was phylogenetically close to the *japonica* group; these results are consistent with those of previous studies (Choi et al., 2018; Civáň et al., 2019). GO enrichment analysis also showed less genomic variation between the Basmati genome and the *aus* group in terms of GO categories. Most of the genes assigned to the three GO categories were mainly involved in metabolic process, cellular process, binding, catalytic activity, cell, and cell part. This functional annotation of genes is consistent with previous findings in rice (Kim et al., 2014; Liu et al., 2017). Additionally, our data showed that the metabolic process involved in the cellular aromatic compound was associated with the common mutated gene cluster (**Figure 5A**) and further analyses revealed that nine genes including *Badh2* gene having alternative allele’s among aromatic group of rice varieties with more than 80% of frequency (**Supplementary Table 8**). However, possible involvement of these genes except *Badh2* remains to be determined for cellular aromatic biosynthesis.

A recent genomic analysis of a population of over 1,000 wild and cultivated rice accessions using genome-wide polymorphisms showed that Basmati rice arose from hybridization between *japonica* and wild rice related to the *aus* group (Civáň et al., 2019). Similarly, our comparative analysis of genome structure, based on genomic variation and GO analysis, showed that the Basmati genome is probably derived mostly from the *aus* and *japonica* groups.

Previously, it was shown that the recessive *fgr* allele encoding *Badh2* carries an 8 bp deletion and three SNPs in the seventh exon, resulting in the fragrant trait in Basmati varieties (Bradbury et al., 2005). Recently, haplotype analysis of the *Badh2* gene and analysis of 2-AP using 242 rice accessions classified two Basmati varieties harboring the wild *Badh2* allele under the *aus* and *indica* groups (Kovach et al., 2009). In this study, our comparative analysis found that both Basmati 370 and Rato Basmati carrying the *badh2.1* allele was consistent with the *badh2.1* allele reported by Kovach et al. (2009). Further, we genotyped the *Badh2* allele in the 30 Basmati varieties using the *Badh2* InDel maker developed by Sakthivel et al. (2009). The results indicated that 22 of the 30 traditional Basmati varieties belonging to the *aromatic* group carry the fragrant-inducing *badh2.1* allele and are more closely related to the *japonica* group. However, eight of the 30 Basmati varieties were harboring the wild *Badh2* allele under the *aus* and *indica* groups (**Supplementary Figure 4**, **Table 1**). Thus, the results of *Badh2* allele genotyping were consistent with those of phylogenetic analysis. We propose that classification of these wild *Badh2* allele carrying Basmati varieties under the *indica* and *aus* groups might results from either natural selection or human error during varietal diversification or germplasm collection.

The success of any crop breeding program depends on the magnitude of genetic variability within the germplasm (Kishor et al., 2016). In this study, although efforts were made to evaluate the agronomic and grain quality traits of 30 traditional Basmati varieties in the experimental field of Seoul National University, most of the Basmati varieties failed to flower in the rice growing season of the temperate region. By contrast, Basmati 370 and a few other wild *Badh2* allele carrying Basmati varieties were flowered, and their agronomic traits were evaluated for further studies in temperate regions (data not shown). Furthermore, agronomic and grain quality trait passport data obtained from the public database Genesys showed wide variation in most of the traits among the 30 traditional Basmati varieties (**Supplementary Table 6**). These finding are in agreement with previous genetic diversity studies in Basmati varieties (Lingaiah et al., 2014; Nirmaladevi et al., 2015). Most of the agronomic and grain quality traits, except L/W ratio, PL, and AC, did not show significant differences among Basmati varieties belonging to the *aromatic* and *indica/aus* groups (**Supplementary Table 7**). AC is an important factor affecting the palatability and grain quality of cooked rice (Tian et al., 2009). Rice grains with low AC (12–20%) are usually glossy, soft, and sticky after cooking, whereas those with high AC (> 25%), generally found in Basmati varieties belonging to the *indica* group, exhibit a dry texture, remain separate, and are less tender upon cooking and become hard upon cooling (Bao et al., 2006).

Hierarchical cluster analysis revealed two major clusters (I and II) among the 30 traditional Basmati varieties, based on agronomic and grain quality traits (**Supplementary Figure 5**). Cluster I comprised varieties from diverse geographical regions, with moderate duration and superior grain qualities. By contrast, cluster II comprised of varieties with late duration and poor grain qualities. These findings are in accordance with a previous study where traditional Basmati varieties with superior agronomic and grain quality traits were grouped in a separate cluster (Roy et al., 2012). Accessions in cluster I with superior agronomic and grain quality could be exploited for the development of improved Basmati varieties in breeding programs.

In conclusion, our study provides a detailed analysis of the Basmati genome structure in comparison with *indica*, *japonica*, and *aus* genomes *via* whole-genome resequencing and genome-wide SNP marker analysis. This data will serve as an important resource for molecular breeding and genetic studies in Basmati rice.

## Data Availability Statement

All relevant raw sequence data are available in the NCBI Short Read Archive (SRA) database under the following BioProject accession numbers: Basmati [PRJNA551546], Dahrdun Basmati [PRJNA551547], and Rato Basmati [PRJNA551548]. 

## Author Contributions

DK and JS conceptualized the study, conducted formal analysis, determined the software for data analysis, and performed data visualization. DK and H-JK curated the data and determined the methodology and resources for this study. JS and JC performed data validation. H-JK acquired the funding and supervised the study. DK, JS, and JC wrote the first draft of the manuscript. All authors reviewed, edited, and approved the final manuscript.

## Funding

This research was funded by the Rural Development Administration through the Next-generation BioGreen 21 Program (Grant No. PJ013165).

## Conflict of Interest

The authors declare that the research was conducted in the absence of any commercial or financial relationships that could be construed as a potential conflict of interest.
