# Supplementary material for: Evaluation of Whole-Genome Sequence, Genetic Diversity, and Agronomic Traits of Basmati Rice (Oryza sativa L.)
Source: Front Genet. 2020 Feb 21;11:86. doi: 10.3389/fgene.2020.00086 (PMC7046879; doi:10.3389/fgene.2020.00086)
Supplement: Supplementary file 1 [file DataSheet_1.docx]

Evaluation of Whole-genome Sequence, Genetic Diversity, and Agronomic Traits of Basmati Rice (*Oryza sativa* L.)

Supplementary Material

# Supplementary Figures and Tables

## Supplementary Figures


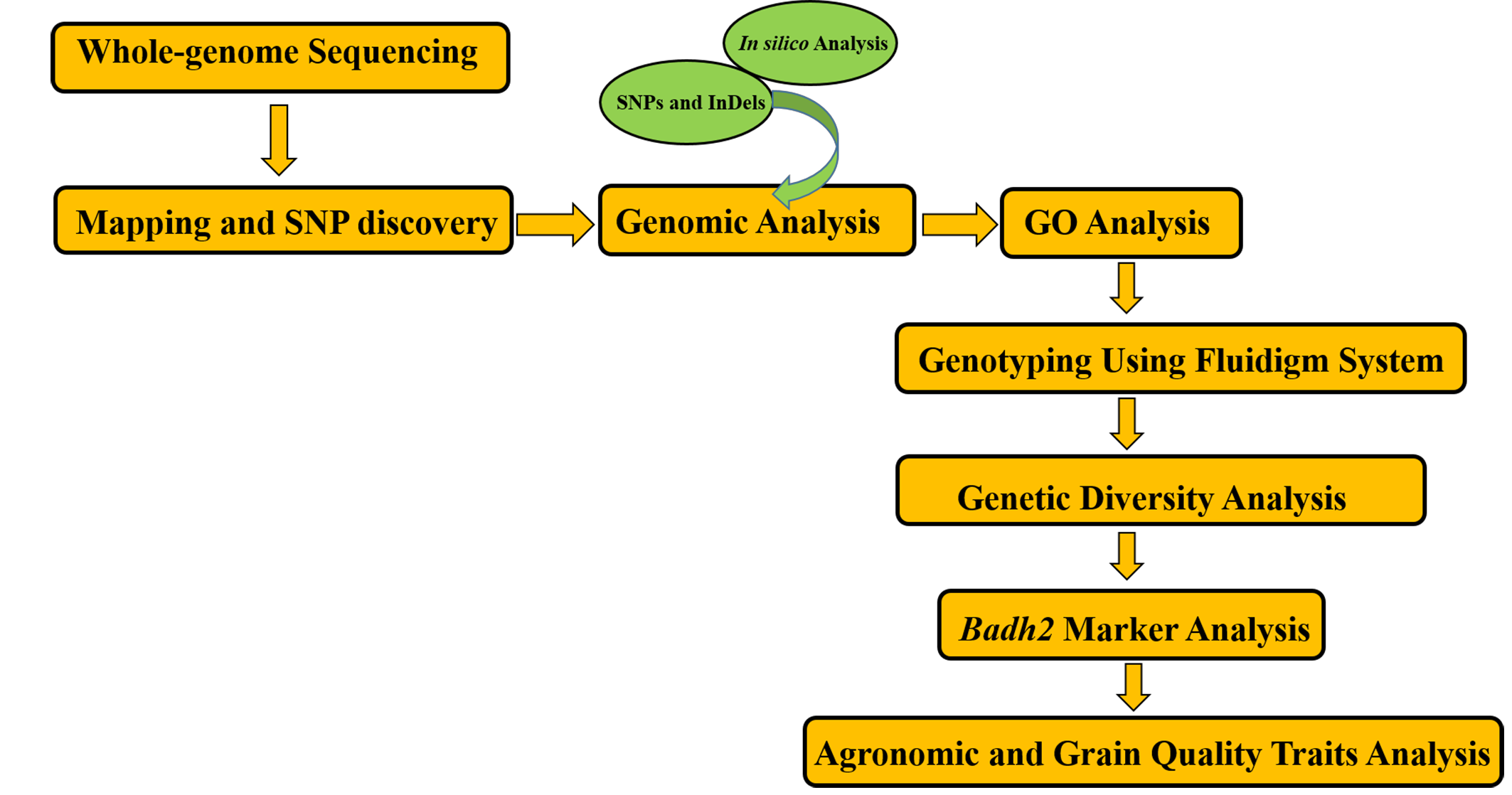


**Supplementary Figure 1.** Overview of experiment. Flow chart indicate work plan of this study


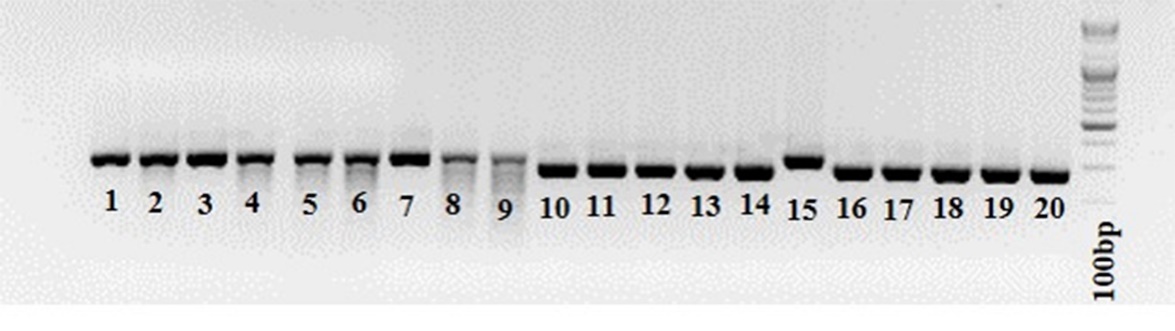


**Supplementary Figure 2.** PCR analysis of InDels associated with the regulation of seed development in rice. Lanes 1–9: Basmati accessions (1, Basmati Kumar; 2, Basmati Gola; 3, Basmati Lamo; 4, Basmati Pahade; 5, Basmati White; 6, Basmati Masino; 7, Basmatiya; 8, Pakistani Basmati; 9, Karnal Basmati). Lanes 10–14: *indica* accessions (10, Milyang_23; 11, IR64; 12, IR8; 13, IR24; 14, Swarna). Lane 15: *aus* accession (Dular). Lanes 16–20: *japonica* accessions (16, Ilpumbyeo; 17, Hopumbyeo; 18, Dongjinbyeo; 19, Koshikari; 20, Samnambyeo).


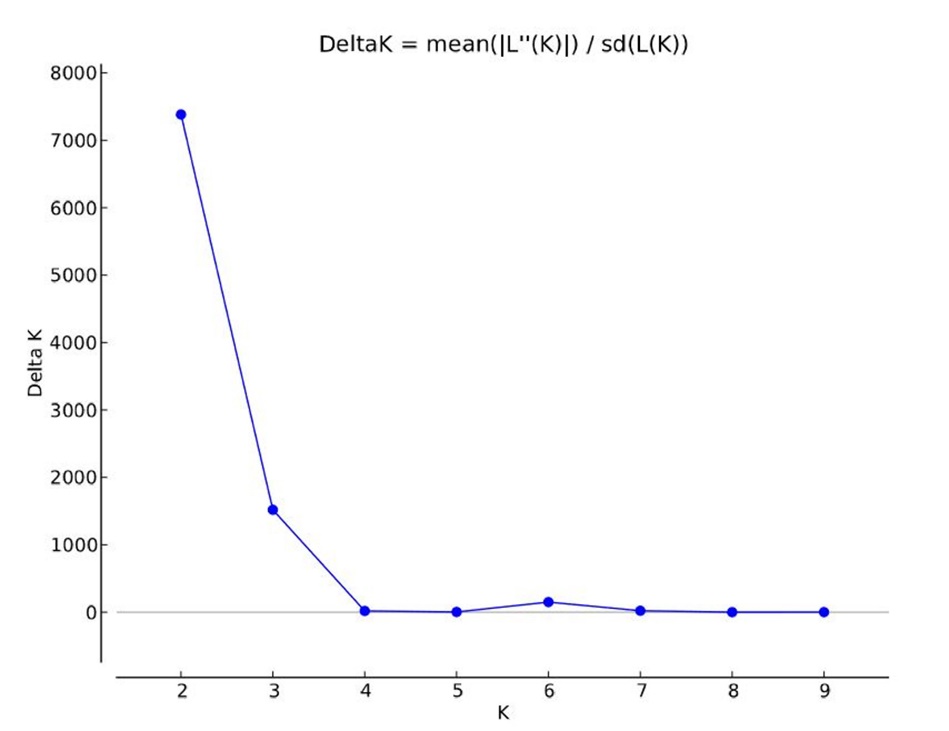


**Supplementary Figure 3.** Delta *K* values for different number of populations (K) assumed based on STRUCTURE analysis.


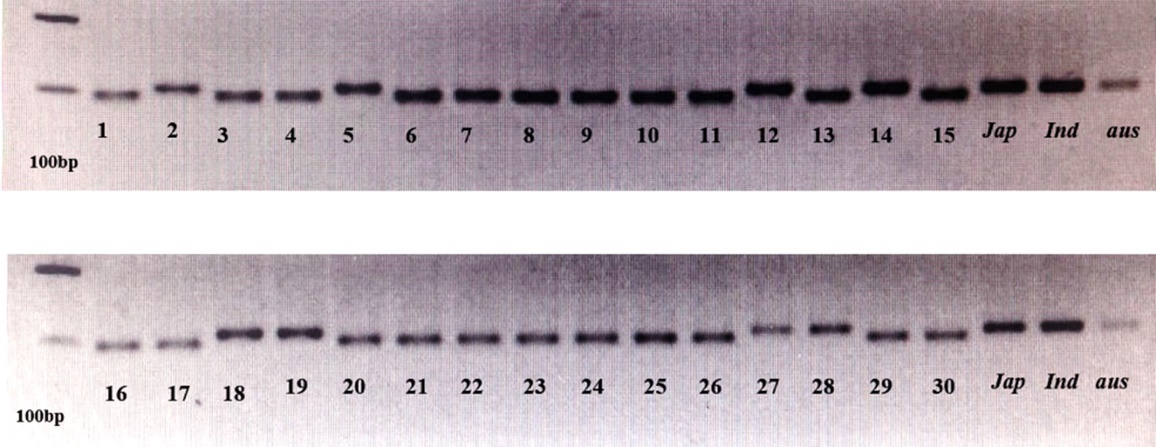


**Supplementary Figure 4**. Analysis of the *fgr* gene-specific InDel marker in Basmati varieties. Lanes 1–30 indicate different Basmati varieties (1, Basmati Dhan; 2, Dheradun Basmati; 3, Basmati Nahan 381; 4, Basmati Sufaid 100; 5, Basmati 140; 6, Basmati 370; 7, Basmati 372; 8, Basmati 377; 9, Deraduni Basmati 321; 10, Kamoh Basmati 392; 11, Sathi Basmati; 12, Basmati Sal; 13, Basmati Kunar; 14, Basmati Kunduz; 15, Basmati Anpjhutte; 16, Basmati Ola; 17, Basmati Lamo, 18, Basmati Masino; 19, Basmati Nokhi; 20, Basmati Pahade; 21, Basmati Red; 22, Basmati White; 23, Basmati Uzarka; 24, Kalo Basmati; 25, Rato Basmati; 26, Basmati Mwea; 27, Dahrdun Basmati; 28, Basmatiya; 29, Pakistani Basmati; 30, Karnal Basmati). *Jap*, Nipponbare; *Ind*, Milyang 23; *aus,* Dular; these were used as check varieties.


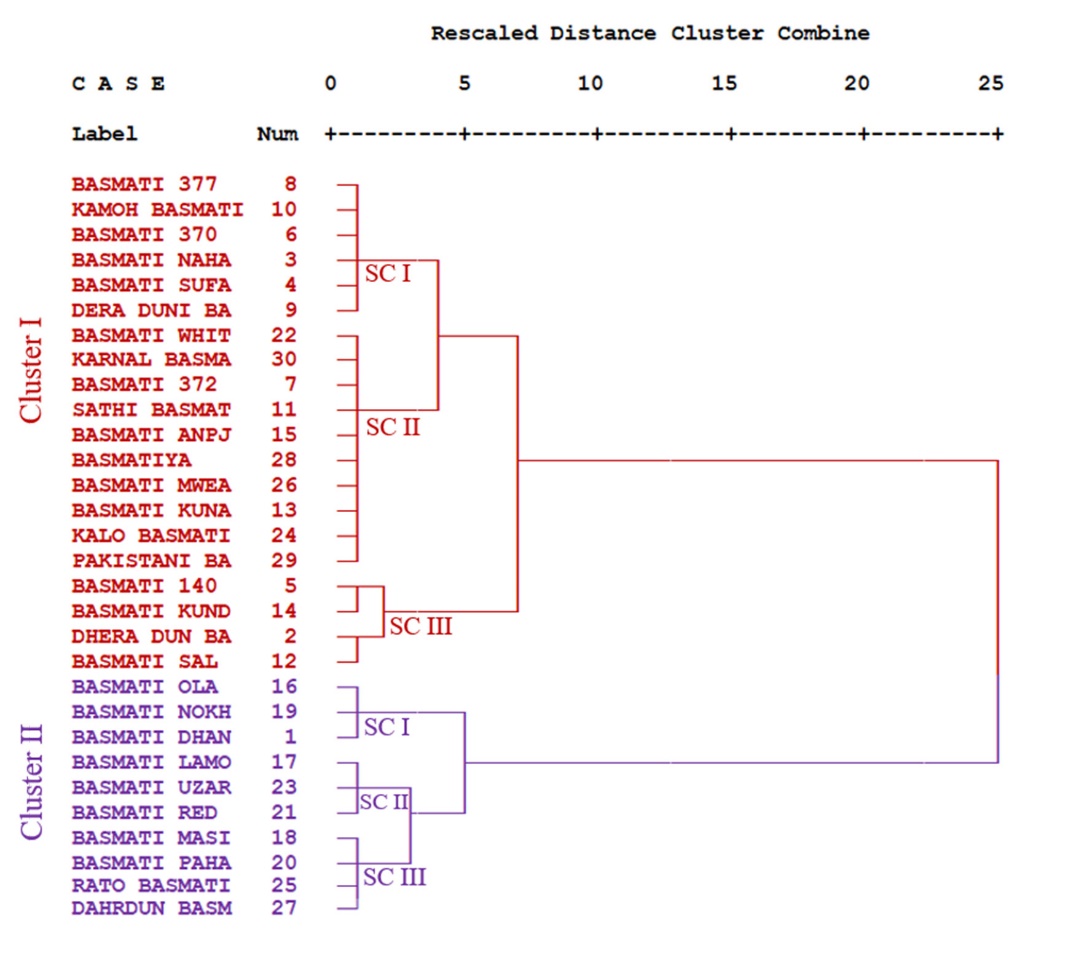


**Supplementary Figure 5.** UPGMA dendrogram of 30 traditional Basmati varieties. Clusters I and II and three subclusters (SC I, II, and III) within each cluster are shown.

## Supplementary Tables

| **Supplementary Table 1. Distribution pattern and number of genome-wide SNPs identified in three Basmati varieties in comparison with Nipponbare, Kasalath, and Zhenshan 97 reference genomes.** | | | | | | | | | | | | | | | | | | | |
| --- | --- | --- | --- | --- | --- | --- | --- | --- | --- | --- | --- | --- | --- | --- | --- | --- | --- | --- | --- |
| **Reference  genomes** | **Chr** | **Non-synonymous** | | | **Synonymous** | | | **Intron** | | | **5' UTR** | | | **3' UTR** | | | **Intergenic** | | |
|  |  | **Basmati 370** | **Dahrdun Basmati** | **Rato Basmati** | **Basmati 370** | **Dahrdun Basmati** | **Rato Basmati** | **Basmati 370** | **Dahrdun Basmati** | **Rato Basmati** | **Basmati 370** | **Dahrdun Basmati** | **Rato Basmati** | **Basmati 370** | **Dahrdun Basmati** | **Rato Basmati** | **Basmati 370** | **Dahrdun Basmati** | **Rato Basmati** |
| Nipponbare  (*japonica*) | 1 | 5,248 | 6,164 | 3,793 | 4,431 | 5,331 | 3,194 | 2,966 | 3,790 | 2,018 | 2,400 | 2,847 | 1,620 | 4,771 | 6,139 | 3,831 | 169,412 | 221,993 | 131,363 |
|  | 2 | 3,426 | 4,618 | 2,415 | 2,876 | 3,883 | 2,057 | 2,189 | 3,312 | 1,598 | 1,534 | 2,559 | 1,116 | 2,780 | 4,456 | 1,976 | 110,139 | 197,885 | 78,903 |
|  | 3 | 3,110 | 4,069 | 1,860 | 2,615 | 3,575 | 1,643 | 2,900 | 3,392 | 1,477 | 1,760 | 2,200 | 1,293 | 3,467 | 4,669 | 2,010 | 162,016 | 193,218 | 82,287 |
|  | 4 | 2,572 | 3,852 | 2,489 | 2,097 | 3,252 | 2,047 | 2,216 | 3,185 | 1,765 | 1,335 | 1,927 | 987 | 2,341 | 3,664 | 1,877 | 105,738 | 158,089 | 95,520 |
|  | 5 | 2,411 | 2,934 | 1,516 | 2,292 | 2,732 | 1,516 | 825 | 1,424 | 603 | 1,136 | 1,399 | 749 | 2,513 | 3,192 | 1,607 | 101,919 | 137,479 | 56,873 |
|  | 6 | 3,546 | 4,063 | 3,979 | 3,242 | 3,580 | 3,585 | 2,497 | 2,410 | 2,668 | 1,641 | 1,607 | 1,747 | 3,236 | 3,839 | 3,661 | 141,606 | 165,966 | 149,764 |
|  | 7 | 3,034 | 3,860 | 2,433 | 2,634 | 3,424 | 2,156 | 2,226 | 3,012 | 1,963 | 1,211 | 1,706 | 921 | 2,246 | 3,094 | 1,734 | 99,163 | 159,366 | 84,547 |
|  | 8 | 2,418 | 3,230 | 2,283 | 2,231 | 2,886 | 1,910 | 1,873 | 1,942 | 1,844 | 1,095 | 1,588 | 1,033 | 2,234 | 3,066 | 1,953 | 106,725 | 143,949 | 102,836 |
|  | 9 | 1,219 | 2,841 | 1,309 | 1,086 | 2,524 | 1,175 | 1,110 | 1,917 | 1,155 | 562 | 1,216 | 615 | 1,219 | 2,670 | 1,361 | 65,960 | 125,132 | 72,786 |
|  | 10 | 2,590 | 3,331 | 2,419 | 2,298 | 2,990 | 2,169 | 2,095 | 2,379 | 1,785 | 997 | 1,242 | 988 | 2,323 | 3,004 | 2,351 | 109,669 | 136,556 | 106,490 |
|  | 11 | 3,712 | 4,314 | 2,722 | 3,015 | 3,550 | 2,149 | 3,124 | 3,392 | 1,395 | 1,238 | 1,461 | 857 | 2,843 | 3,199 | 1,945 | 131,827 | 154,927 | 75,490 |
|  | 12 | 2,095 | 2,702 | 1,266 | 1,950 | 2,454 | 1,150 | 1,419 | 1,574 | 1,064 | 978 | 1,277 | 717 | 1,856 | 2,409 | 1,267 | 100,921 | 128,141 | 81,510 |
| Kasalath  (*aus*) | 1 | 1,892 | 1,849 | 1,787 | 1,546 | 1,591 | 1,525 | 4,352 | 4,600 | 4,307 | 313 | 298 | 298 | 610 | 673 | 706 | 160,319 | 160,045 | 176,258 |
|  | 2 | 1,506 | 1,399 | 1,503 | 1,244 | 1,101 | 1,185 | 3,617 | 3,292 | 3,708 | 220 | 256 | 220 | 438 | 513 | 438 | 137,884 | 122,060 | 152,860 |
|  | 3 | 1,159 | 1,156 | 1,471 | 830 | 915 | 1,131 | 3,008 | 3,115 | 3,841 | 176 | 193 | 242 | 391 | 411 | 530 | 121,546 | 125,563 | 173,085 |
|  | 4 | 1,072 | 1,305 | 1,322 | 831 | 1,071 | 941 | 2,372 | 3,131 | 3,011 | 121 | 194 | 127 | 294 | 366 | 290 | 104,443 | 106,296 | 135,002 |
|  | 5 | 920 | 846 | 1,288 | 665 | 602 | 992 | 2,159 | 2,153 | 2,770 | 125 | 123 | 170 | 262 | 296 | 419 | 119,731 | 111,650 | 160,044 |
|  | 6 | 1,480 | 1,158 | 1,321 | 1,044 | 872 | 941 | 3,491 | 2,647 | 3,388 | 194 | 109 | 165 | 420 | 333 | 386 | 144,268 | 124,359 | 128,515 |
|  | 7 | 1,238 | 860 | 1,407 | 1,030 | 742 | 1,123 | 2,760 | 1,980 | 2,921 | 196 | 118 | 199 | 364 | 257 | 378 | 133,515 | 91,878 | 143,689 |
|  | 8 | 1,078 | 793 | 1,033 | 773 | 631 | 696 | 2,148 | 1,971 | 1,993 | 124 | 129 | 110 | 285 | 243 | 262 | 110,197 | 84,198 | 106,084 |
|  | 9 | 921 | 730 | 884 | 742 | 603 | 682 | 2,164 | 1,735 | 2,098 | 120 | 105 | 112 | 211 | 275 | 198 | 106,442 | 78,478 | 99,153 |
|  | 10 | 607 | 627 | 791 | 480 | 472 | 630 | 970 | 1,230 | 1,264 | 95 | 64 | 103 | 205 | 180 | 228 | 65,831 | 70,014 | 83,974 |
|  | 11 | 1,443 | 1,805 | 1,334 | 1,165 | 1,459 | 992 | 2,967 | 3,442 | 3,096 | 79 | 151 | 107 | 220 | 338 | 235 | 108,187 | 108,388 | 114,741 |
|  | 12 | 688 | 881 | 655 | 555 | 700 | 556 | 1,581 | 1,548 | 1,394 | 113 | 136 | 76 | 235 | 214 | 155 | 78,587 | 92,626 | 87,941 |
| Zhenshan 97  (*indica*) | 1 | 10,858 | 8,374 | 11,101 | 7,472 | 6,036 | 7,766 | 10,158 | 8,391 | 11,288 | 5,593 | 2,761 | 3,869 | 2,952 | 4,698 | 7,125 | 114,920 | 90,986 | 130,196 |
|  | 2 | 11,087 | 3,169 | 11,608 | 7,448 | 2,396 | 7,592 | 10,291 | 3,097 | 10,028 | 3,137 | 1,050 | 3,344 | 5,968 | 2,128 | 6,201 | 132,799 | 40,530 | 139,318 |
|  | 3 | 7,219 | 4,014 | 9,786 | 4,609 | 2,530 | 6,141 | 6,090 | 3,230 | 8,692 | 2,554 | 1,176 | 3,431 | 4,366 | 2,104 | 5,670 | 94,436 | 48,711 | 123,813 |
|  | 4 | 5,738 | 5,218 | 9,662 | 4,064 | 3,531 | 6,450 | 6,578 | 5,203 | 8,997 | 1,840 | 1,298 | 2,444 | 3,671 | 2,690 | 4,631 | 65,348 | 56,164 | 102,114 |
|  | 5 | 5,603 | 2,835 | 8,831 | 3,459 | 1,842 | 5,892 | 4,483 | 2,364 | 7,542 | 1,351 | 768 | 2,556 | 2,195 | 1,241 | 4,572 | 57,713 | 33,408 | 103,844 |
|  | 6 | 9,583 | 3,952 | 9,604 | 6,595 | 2,586 | 6,521 | 9,876 | 3,794 | 9,926 | 2,499 | 1,086 | 2,395 | 4,853 | 2,015 | 5,004 | 114,062 | 45,693 | 113,032 |
|  | 7 | 8,552 | 3,777 | 9,855 | 5,498 | 2,562 | 6,468 | 8,287 | 3,600 | 8,922 | 1,910 | 919 | 2,300 | 4,121 | 1,812 | 4,858 | 98,178 | 35,660 | 114,192 |
|  | 8 | 5,259 | 2,404 | 6,002 | 3,047 | 1,433 | 3,859 | 4,980 | 2,139 | 5,847 | 707 | 415 | 1,102 | 1,339 | 845 | 1,966 | 92,703 | 57,192 | 102,645 |
|  | 9 | 6,836 | 5,647 | 7,658 | 4,492 | 3,578 | 5,027 | 6,052 | 5,105 | 6,686 | 1,717 | 1,143 | 1,917 | 3,135 | 2,260 | 3,439 | 72,811 | 57,063 | 82,153 |
|  | 10 | 8,049 | 3,925 | 9,014 | 5,312 | 2,662 | 5,920 | 7,328 | 3,586 | 7,998 | 1,434 | 764 | 1,575 | 2,900 | 1,475 | 3,151 | 81,726 | 39,240 | 91,794 |
|  | 11 | 8,349 | 5,380 | 11,479 | 5,563 | 3,764 | 7,276 | 8,826 | 4,800 | 11,574 | 1,465 | 952 | 1,853 | 3,426 | 2,160 | 3,730 | 80,458 | 48,647 | 104,508 |
|  | 12 | 7,960 | 5,904 | 7,318 | 5,185 | 3,657 | 4,680 | 6,553 | 4,841 | 6,313 | 1,778 | 1,139 | 1,573 | 3,391 | 1,966 | 2,865 | 88,334 | 60,444 | 80,751 |

| **Supplementary Table 2.** Distribution pattern and number of genome-wide InDels and SNPs in Basmati varieties compared with Nipponbare, Kasalath, and Zhenshan 97 reference genomes. | | | | | | | | | | | |
| --- | --- | --- | --- | --- | --- | --- | --- | --- | --- | --- | --- |
| **Reference  genomes** | **Chr** | **InDels** | | | **SNPs** | | | **Total nucleotide polymorphisms** | | | |
|  |  | **Basmati 370** | **Dahrdun Basmati** | **Rato Basmati** | **Basmati 370** | **Dahrdun Basmati** | **Rato Basmati** | **Basmati 370** | **Dahrdun Basmati** | **Rato Basmati** |  |
| Nipponbare | 1 | 28,648 | 223,801 | 132,524 | 160,580 | 22,463 | 13,295 | 189,228 | 246,264 | 145,819 |  |
|  | 2 | 111,124 | 199,448 | 79,594 | 11,820 | 17,265 | 8,471 | 122,944 | 216,713 | 88,065 |  |
|  | 3 | 163,216 | 194,686 | 83,026 | 12,652 | 16,437 | 7,544 | 175,868 | 211,123 | 90,570 |  |
|  | 4 | 106,583 | 159,390 | 96,255 | 9,716 | 14,579 | 8,430 | 116,299 | 173,969 | 104,685 |  |
|  | 5 | 102,637 | 138,406 | 10,369 | 8,459 | 10,754 | 52,495 | 111,096 | 149,160 | 62,864 |  |
|  | 6 | 142,800 | 167,130 | 151,022 | 12,968 | 14,335 | 14,382 | 155,768 | 181,465 | 165,404 |  |
|  | 7 | 100,093 | 160,583 | 85,350 | 10,421 | 13,879 | 8,404 | 110,514 | 174,462 | 93,754 |  |
|  | 8 | 107,484 | 144,926 | 103,540 | 9,092 | 11,735 | 8,319 | 116,576 | 156,661 | 111,859 |  |
|  | 9 | 66,357 | 125,937 | 73,207 | 4,799 | 10,363 | 5,194 | 71,156 | 136,300 | 78,401 |  |
|  | 10 | 110,474 | 137,499 | 140,532 | 9,498 | 12,003 | 8,970 | 119,972 | 149,502 | 116,202 |  |
|  | 11 | 67,523 | 156,104 | 162,369 | 78,236 | 14,739 | 8,474 | 145,759 | 170,843 | 84,558 |  |
|  | 12 | 101,620 | 128,970 | 133,576 | 7,599 | 9,587 | 4,981 | 109,219 | 138,557 | 81,510 |  |
| Kasalath | 1 | 26,852 | 26,741 | 28,673 | 142,180 | 142,315 | 156,208 | 169,032 | 169,056 | 184,881 |  |
|  | 2 | 25,105 | 24,213 | 27,224 | 119,804 | 104,408 | 132,690 | 144,909 | 128,621 | 159,914 |  |
|  | 3 | 23,669 | 25,095 | 30,282 | 103,441 | 106,258 | 150,018 | 127,110 | 131,353 | 180,300 |  |
|  | 4 | 19,529 | 19,690 | 22,661 | 89,604 | 92,673 | 118,032 | 109,133 | 112,363 | 140,693 |  |
|  | 5 | 19,677 | 19,060 | 26,079 | 104,185 | 96,610 | 139,604 | 123,862 | 115,670 | 165,683 |  |
|  | 6 | 24,898 | 21,576 | 22,606 | 125,999 | 107,902 | 112,110 | 150,897 | 129,478 | 134,716 |  |
|  | 7 | 22,261 | 17,311 | 23,674 | 116,842 | 78,524 | 126,043 | 139,103 | 95,835 | 149,717 |  |
|  | 8 | 19,100 | 15,474 | 18,472 | 95,505 | 72,491 | 91,706 | 114,605 | 87,965 | 110,178 |  |
|  | 9 | 17,236 | 14,242 | 16,181 | 93,364 | 67,684 | 86,946 | 110,600 | 81,926 | 103,127 |  |
|  | 10 | 12,429 | 12,814 | 14,172 | 55,759 | 59,773 | 72,818 | 68,188 | 72,587 | 86,990 |  |
|  | 11 | 18,476 | 18,723 | 18,723 | 95,585 | 96,859 | 101,782 | 114,061 | 115,582 | 120,505 |  |
|  | 12 | 14,077 | 12,751 | 15,661 | 67,682 | 79,875 | 75,116 | 81,759 | 92,626 | 90,777 |  |
| Zhenshan 97 | 1 | 22,849 | 18,947 | 26,716 | 129,104 | 102,299 | 144,629 | 151,953 | 121,246 | 171,345 |  |
|  | 2 | 25,402 | 8,807 | 26,612 | 145,328 | 43,563 | 151,479 | 170,730 | 52,370 | 178,091 |  |
|  | 3 | 18,475 | 9,464 | 23,271 | 100,799 | 52,301 | 134,262 | 119,274 | 61,765 | 157,533 |  |
|  | 4 | 12,993 | 11,090 | 18,385 | 74,246 | 63,014 | 115,913 | 87,239 | 74,104 | 134,298 |  |
|  | 5 | 10,568 | 6,750 | 19,904 | 64,236 | 35,708 | 113,333 | 74,804 | 42,458 | 133,237 |  |
|  | 6 | 21,662 | 8,961 | 21,477 | 125,806 | 50,165 | 125,005 | 147,468 | 59,126 | 146,482 |  |
|  | 7 | 17,627 | 7,197 | 20,684 | 108,919 | 41,133 | 125,911 | 126,546 | 48,330 | 146,595 |  |
|  | 8 | 15,312 | 9,535 | 17,551 | 92,723 | 54,893 | 103,870 | 108,035 | 64,428 | 121,421 |  |
|  | 9 | 13,335 | 10,488 | 15,031 | 81,708 | 64,308 | 91,849 | 95,043 | 74,796 | 106,880 |  |
|  | 10 | 14,864 | 7,305 | 16,096 | 91,885 | 44,347 | 103,356 | 106,749 | 51,652 | 119,452 |  |
|  | 11 | 15,832 | 10,146 | 19,048 | 92,255 | 55,557 | 121,372 | 108,087 | 65,703 | 140,420 |  |
|  | 12 | 16,600 | 10,841 | 14,720 | 96,601 | 67,110 | 88,780 | 113,201 | 77,951 | 103,500 |  |

| **Supplementary Table 3.** Unique SNPs and InDels identified in Basmati varieties via *in silico* analysis. | | | | | | | | | | | | | | |
| --- | --- | --- | --- | --- | --- | --- | --- | --- | --- | --- | --- | --- | --- | --- |
| **Nucleotide variation** | | | **Chr** | | **Position (bp)** | | **Gene ID** | | **Variant sequence** | | **Variant type or location** | | **Gene function** | |
| SNPs | Substitution | 1 | | 734,328 | | *Os01g0113500* | | G**/**A | | Non_synonymous | | Receptor like kinase | |  |
|  | Substitution | 1 | | 734,333 | | *Os01g0113500* | | C**/**T | | Synonymous | | Receptor like kinase | |  |
|  | Substitution | 1 | | 734,337 | | *Os01g0113500* | | G**/**T | | Non_synonymous | | Receptor like kinase | |  |
|  | Substitution | 1 | | 734,338 | | *Os01g0113500* | | C/T | | Non_synonymous | | Receptor like kinase | |  |
|  | Substitution | 1 | | 734,343 | | *Os01g0113500* | | A**/**C | | Non_synonymous | | Receptor like kinase | |  |
|  | Substitution | 1 | | 734,352 | | *Os01g0113500* | | C**/**G | | Non_synonymous | | Receptor like kinase | |  |
|  | Substitution | 1 | | 734,353 | | *Os01g0113500* | | C**/**T | | Non_synonymous | | Receptor like kinase | |  |
|  | Substitution | 1 | | 734,366 | | *Os01g0113500* | | T**/**A | | Non_synonymous | | Receptor like kinase | |  |
|  | Substitution | 1 | | 734,369 | | *Os01g0113500* | | C**/**G | | Non_synonymous | | Receptor like kinase | |  |
|  | Substitution | 2 | | 23,605,555 | | *Os02g0602900* | | C**/**T | | UTR_5_Prime | | Conserved hypothetical protein. | |  |
|  | Substitution | 4 | | 14,493,128 | | *Os04g0316800* | | C**/**G | | Non_synonymous | | Similar to OSIGBa0113K06.4 proteins. | |  |
|  | Substitution | 4 | | 14,693,794 | | *Os04g0319800* | | T**/**G | | Synonymous | | Similar to Cytokinin-O-glucosyltransferase 2 | |  |
|  | Substitution | 5 | | 15,385,095 | | *Os05g0329700* | | T**/**C | | Non_synonymous | | Similar to Prolamin. | |  |
|  | Substitution | 6 | | 1,344,310 | | *Os06g0124900* | | C**/**G | | UTR_3_Prime | | Conserved hypothetical protein. | |  |
|  | Substitution | 6 | | 22,799,559 | | *Os06g0583400* | | C**/**T | | Intron | | Class-I type histone deacetylase, Seedling root growth | |  |
|  | Substitution | 8 | | 6,626,996 | | *Os08g0214233* | | A**/**G | | UTR_3_Prime | | Conserved hypothetical protein. | |  |
|  | Substitution | 9 | | 831,211 | | *Os09g0109150* | | C**/**A | | Synonymous | | Conserved hypothetical protein. | |  |
|  | Substitution | 10 | | 7,133,301 | | *Os10g0200800* | | A**/**G | | Intron | | Similar to Phosphatidylinositol glycan synthesis class F protein | |  |
|  | Substitution | 11 | | 22,825,265 | | *Os11g0598300* | | G**/**T | | Non_synonymous | | NB-ARC domain containing protein. | |  |
|  | Substitution | 11 | | 260,90,487 | | *Os11g0653300* | | C**/**T | | Non_synonymous | | Conserved hypothetical protein. | |  |
| InDels | Insertion | 1 | | 23,113,859 | | *Os01G0591500* | | C**/**CGGCGGCGGCGGCGGCGGG | | Intron | | Non-protein coding transcript | |  |
|  | Insertion | 1 | | 40,560,556 | | *Os01G0925100* | | A**/**AAAAGGACCAGATTTGGCTGC | | UTR_3_Prime | | Conserved hypothetical protein | |  |
|  | Insertion | 4 | | 2,522,158 | | *Os04G0137550* | | A**/**AAATCAAGAACAATGAT | | UTR_5_Prime | | hypothetical protein | |  |
|  | Deletion | 5 | | 2,870,305 | | *Os05G0150100* | | AATATAAGACTTTTTAGCATTACTCACATTC**/**A | | UTR_3_Prime | | Conserved hypothetical protein | |  |
|  | Insertion | 5 | | 29,593,376 | | *Os05G0594200* | | T**/**TGAGAGAGAGAGAGAGAGAGAGGAGA | | UTR_5_Prime | | Similar to Cation/proton exchanger 1a. | |  |
|  | Insertion | 7 | | 26,745,874 | | *Os07G0642400* | | T**/**TGTGCTGCAGCAGTTG | | Intron | | Similar to chromatin remodeling complex | |  |
|  | Insertion | 9 | | 1,989,082 | | *Os09G0125766* | | G**/**GTTGTAGTTTACCTCTAGCCAAGAACAA | | Intron | | Hypothetical gene | |  |
|  | Insertion | 10 | | 2,425,049 | | *Os10G0139300* | | C**/**CCAGTCAATTAACCATATACTGAGT | | Intron | | Regulation of seed development | |  |
|  | Insertion | 10 | | 6,756,982 | | *Os10G0197300* | | A**/**ACCGCCGACTCGTGTTT | | UTR_3_Prime | | Conserved hypothetical gene | |  |
|  | Insertion | 10 | | 17,330,627 | | *Os10G0468950* | | C**/**CTCCTCCGCCCCCCCTCT | | UTR_5_Prime | | hypothetical protein | |  |
|  | Insertion | 12 | | 7,489,742 | | *Os12G0236500* | | T**/**TTGGCTCGTCCTGACTCGA | | UTR_5_Prime | | Similar to Aspartyl aminopeptidase-like protein. | |  |

**Supplementary Table 4.** Distribution of the 190-plex *japonica/indica* SNP set in 60 rice varieties

| **Chromosome** | **1** | **2** | **3** | **4** | **5** | **6** | **7** | **8** | **9** | **10** | **11** | **12** | **Total** |
| --- | --- | --- | --- | --- | --- | --- | --- | --- | --- | --- | --- | --- | --- |
| Number of SNPs | 23 | 17 | 20 | 17 | 16 | 19 | 16 | 14 | 11 | 10 | 14 | 13 | 190 |
| Average physical interval (Mb) | 2.01 | 2.15 | 1.96 | 2.23 | 1.98 | 1.75 | 2.01 | 2.12 | 2.10 | 2.57 | 2.34 | 2.28 | 2.09 |
| Physical coverage (%) | 98.2 | 99.5 | 99.6 | 98.8 | 98.7 | 97.9 | 99.5 | 96.4 | 96.4 | 97.4 | 96.2 | 99.0 | 98.2 |

**Supplementary Table 5.** Genotype summary of the 190-plex *indica/japonica* SNP set in 60 rice varieties

| **No.** | **Chr.** | **SNP ID** | **Position (IRGSP.v5)** | **MAF*^a^*** | **No. of alleles** | **Heterozygosity** | **PIC*^b^*** |
| --- | --- | --- | --- | --- | --- | --- | --- |
| 1 | 1 | id1000223 | 422,620 | 0.6833 | 2 | 0.0000 | 0.3391 |
| 2 | 1 | cbm0103.4 | 3,494,000 | 0.7000 | 2 | 0.0000 | 0.3318 |
| 3 | 1 | id1004256 | 5,332,883 | 0.6000 | 2 | 0.0000 | 0.3648 |
| 4 | 1 | ad01003587 | 7,435,035 | 0.5833 | 2 | 0.0000 | 0.3680 |
| 5 | 1 | id1007185 | 9,670,523 | 0.7333 | 2 | 0.0000 | 0.3146 |
| 6 | 1 | ad01005318 | 12,048,871 | 0.7167 | 2 | 0.0000 | 0.3236 |
| 7 | 1 | id1009557 | 14,573,814 | 0.6833 | 2 | 0.0000 | 0.3391 |
| 8 | 1 | id1010652 | 19,264,312 | 0.8167 | 2 | 0.0000 | 0.2546 |
| 9 | 1 | ah01001478 | 22,138,518 | 0.7167 | 2 | 0.0000 | 0.3236 |
| 10 | 1 | SaF-CT | 24,007,114 | 0.6833 | 2 | 0.0000 | 0.3391 |
| 11 | 1 | id1012784 | 24,199,706 | 0.6833 | 2 | 0.0000 | 0.3391 |
| 12 | 1 | ah01001843 | 26,813,843 | 0.5667 | 2 | 0.0000 | 0.3705 |
| 13 | 1 | Rd-GA | 27,013,911 | 0.6667 | 2 | 0.0000 | 0.3457 |
| 14 | 1 | id1015984 | 29,381,536 | 0.7500 | 2 | 0.0000 | 0.3047 |
| 15 | 1 | id1018870 | 33,063,515 | 0.6000 | 2 | 0.0000 | 0.3648 |
| 16 | 1 | ad01015967 | 34,978,783 | 0.6000 | 2 | 0.0000 | 0.3648 |
| 17 | 1 | id1022407 | 37,298,045 | 0.6167 | 2 | 0.0000 | 0.3610 |
| 18 | 1 | qSH1-TG | 38,191,474 | 0.8500 | 2 | 0.0000 | 0.2225 |
| 19 | 1 | SD1-GA | 40,114,083 | 0.6167 | 2 | 0.0000 | 0.3610 |
| 20 | 1 | id1024836 | 40,892,591 | 0.7500 | 2 | 0.0000 | 0.3047 |
| 21 | 1 | P1193 | 42,494,829 | 0.7333 | 2 | 0.0000 | 0.3146 |
| 22 | 1 | ad01020824 | 43,613,762 | 0.7667 | 2 | 0.0000 | 0.2938 |
| 23 | 1 | id1028304 | 44,672,306 | 0.7833 | 2 | 0.0000 | 0.2818 |
| 24 | 2 | id2000007 | 9,619 | 0.8167 | 2 | 0.0000 | 0.2546 |
| 25 | 2 | ad02000512 | 2,406,169 | 0.8333 | 2 | 0.0000 | 0.2392 |
| 26 | 2 | id2002293 | 4,361,466 | 0.5500 | 2 | 0.0000 | 0.3725 |
| 27 | 2 | ah02000407 | 6,739,079 | 0.7333 | 2 | 0.0000 | 0.3146 |
| 28 | 2 | id2004617 | 9,582,164 | 0.5167 | 2 | 0.0000 | 0.3747 |
| 29 | 2 | id2005923 | 14,795,354 | 0.7333 | 2 | 0.0000 | 0.3146 |
| 30 | 2 | cmb0219.1 | 19,193,676 | 0.8000 | 3 | 0.0000 | 0.2827 |
| 31 | 2 | id2007512 | 20,027,495 | 0.5833 | 2 | 0.0000 | 0.3680 |
| 32 | 2 | ah02001499 | 21,968,850 | 0.5833 | 2 | 0.0000 | 0.3680 |
| 33 | 2 | id2009889 | 24,835,540 | 0.7167 | 2 | 0.0000 | 0.3236 |
| 34 | 2 | ad02011845 | 26,817,689 | 0.8167 | 2 | 0.0000 | 0.2546 |
| 35 | 2 | id2012773 | 29,130,590 | 0.7500 | 2 | 0.0000 | 0.3047 |
| 36 | 2 | ae02004877 | 30,861,574 | 0.7833 | 2 | 0.0000 | 0.2818 |
| 37 | 2 | id2014575 | 33,610,265 | 0.6333 | 2 | 0.0000 | 0.3566 |
| 38 | 2 | cmb0235.4 | 35,456,937 | 0.7167 | 2 | 0.0000 | 0.3236 |
| 39 | 2 | id2016199 | 36,199,294 | 0.8167 | 2 | 0.0000 | 0.2546 |
| 40 | 2 | cmb0236.6 | 36,626,894 | 0.6833 | 2 | 0.0000 | 0.3391 |
| 41 | 3 | ad03000001 | 25,505 | 0.7833 | 2 | 0.0000 | 0.2818 |
| 42 | 3 | id3000695 | 1,086,244 | 0.6500 | 2 | 0.0000 | 0.3515 |
| 43 | 3 | ah03000403 | 3,524,497 | 0.7167 | 2 | 0.0000 | 0.3236 |
| 44 | 3 | id3003462 | 5,920,066 | 0.7333 | 2 | 0.0000 | 0.3146 |
| 45 | 3 | ah03000736 | 7,951,736 | 0.7000 | 2 | 0.0000 | 0.3318 |
| 46 | 3 | id3005168 | 10,023,081 | 0.5333 | 2 | 0.0000 | 0.3739 |
| 47 | 3 | ah03001094 | 12,321,614 | 0.7000 | 2 | 0.0000 | 0.3318 |
| 48 | 3 | id3007541 | 15,548,178 | 0.8000 | 2 | 0.0000 | 0.2688 |
| 49 | 3 | GS3-CA | 17,365,441 | 0.6167 | 2 | 0.0000 | 0.3610 |
| 50 | 3 | id3009433 | 20,673,413 | 0.5917 | 2 | 0.0167 | 0.3665 |
| 51 | 3 | id3010700 | 24,305,190 | 0.5500 | 2 | 0.0000 | 0.3725 |
| 52 | 3 | ad03013905 | 25,848,924 | 0.5833 | 2 | 0.0000 | 0.3680 |
| 53 | 3 | ad03014175 | 27,135,408 | 0.8500 | 2 | 0.0000 | 0.2225 |
| 54 | 3 | dd3000535 | 28,481,283 | 0.8833 | 2 | 0.0000 | 0.1849 |
| 55 | 3 | ae03006317 | 30,699,147 | 0.7333 | 2 | 0.0000 | 0.3146 |
| 56 | 3 | Hd6-AT | 32,410,973 | 0.8167 | 2 | 0.0000 | 0.2546 |
| 57 | 3 | id3015453 | 32,981,871 | 0.5500 | 2 | 0.0000 | 0.3725 |
| 58 | 3 | ah03002520 | 35,132,848 | 0.6333 | 2 | 0.0000 | 0.3566 |
| 59 | 3 | cmb0336.5 | 36,572,765 | 0.6500 | 2 | 0.0000 | 0.3515 |
| 60 | 3 | id3018439 | 37,181,952 | 0.7500 | 2 | 0.0000 | 0.3047 |
| 61 | 4 | P0610_1 | 227,136 | 0.5833 | 2 | 0.0000 | 0.3680 |
| 62 | 4 | id4001096 | 2,456,955 | 0.7667 | 2 | 0.0000 | 0.2938 |
| 63 | 4 | id4002718 | 6,997,191 | 0.5833 | 3 | 0.0000 | 0.4453 |
| 64 | 4 | id4003524 | 11,312,866 | 0.7833 | 3 | 0.0000 | 0.2967 |
| 65 | 4 | id4004185 | 14,389,955 | 0.7333 | 2 | 0.0000 | 0.3146 |
| 66 | 4 | cmb0417.4 | 17,499,419 | 0.6333 | 3 | 0.0000 | 0.4279 |
| 67 | 4 | cmb0418.8 | 18,813,976 | 0.7667 | 3 | 0.0000 | 0.3419 |
| 68 | 4 | id4005704 | 19,826,416 | 0.7333 | 2 | 0.0000 | 0.3146 |
| 69 | 4 | cmb0420.7 | 20,730,844 | 0.5000 | 2 | 0.0000 | 0.3750 |
| 70 | 4 | cmb0422.7 | 22,750,935 | 0.7667 | 3 | 0.0000 | 0.3095 |
| 71 | 4 | id4007882 | 24,379,789 | 0.7917 | 3 | 0.0500 | 0.3110 |
| 72 | 4 | ad04009559 | 26,443,283 | 0.8167 | 2 | 0.0000 | 0.2546 |
| 73 | 4 | ah04001252 | 28,788,134 | 0.7000 | 2 | 0.0000 | 0.3318 |
| 74 | 4 | id4009823 | 30,014,981 | 0.7167 | 2 | 0.0000 | 0.3236 |
| 75 | 4 | cmb0432.2 | 32,268,324 | 0.7833 | 2 | 0.0000 | 0.2818 |
| 76 | 4 | cmb0434.1 | 34,108,743 | 0.7500 | 2 | 0.0000 | 0.3047 |
| 77 | 4 | id4012434 | 35,843,455 | 0.7167 | 2 | 0.0000 | 0.3236 |
| 78 | 5 | id5000043 | 101,950 | 0.5167 | 2 | 0.0000 | 0.3747 |
| 79 | 5 | cmb0500.9 | 974,535 | 0.7750 | 3 | 0.0167 | 0.3032 |
| 80 | 5 | cmb0501.9 | 1,907,768 | 0.5333 | 2 | 0.0000 | 0.3739 |
| 81 | 5 | GS5-GA | 3,444,074 | 0.7667 | 2 | 0.0000 | 0.2938 |
| 82 | 5 | id5002497 | 4,734,427 | 0.6833 | 2 | 0.0000 | 0.3391 |
| 83 | 5 | qSW5-AG | 5,361,402 | 0.5167 | 2 | 0.0000 | 0.3747 |
| 84 | 5 | id5004086 | 8,028,641 | 0.6167 | 2 | 0.0000 | 0.3610 |
| 85 | 5 | cmb0511.1 | 11,127,260 | 0.8000 | 2 | 0.0000 | 0.2688 |
| 86 | 5 | id5005882 | 14,387,882 | 0.8000 | 2 | 0.0000 | 0.2688 |
| 87 | 5 | ah05000909 | 17,422,595 | 0.5500 | 2 | 0.0000 | 0.3725 |
| 88 | 5 | id5008218 | 20,019,740 | 0.6167 | 2 | 0.0000 | 0.3610 |
| 89 | 5 | ad05008445 | 22,039,255 | 0.6833 | 2 | 0.0000 | 0.3391 |
| 90 | 5 | id5010886 | 24,121,914 | 0.6000 | 2 | 0.0000 | 0.3648 |
| 91 | 5 | cmb0526.3 | 26,356,605 | 0.6000 | 3 | 0.0000 | 0.4910 |
| 92 | 5 | id5014265 | 28,700,370 | 0.7333 | 2 | 0.0000 | 0.3146 |
| 93 | 5 | cmb0529.7 | 29,798,006 | 0.8500 | 2 | 0.0000 | 0.2225 |
| 94 | 6 | id6000073 | 243,274 | 0.6833 | 2 | 0.0000 | 0.3391 |
| 95 | 6 | GS6-TG | 1,464,867 | 0.6000 | 2 | 0.0000 | 0.3648 |
| 96 | 6 | WAXY-TG | 1,764,762 | 0.7500 | 2 | 0.0000 | 0.3047 |
| 97 | 6 | id6003373 | 4,756,949 | 0.5500 | 2 | 0.0000 | 0.3725 |
| 98 | 6 | cmb0605.5 | 5,593,526 | 0.8500 | 2 | 0.0000 | 0.2225 |
| 99 | 6 | S5-TC | 5,760,512 | 0.8000 | 2 | 0.0000 | 0.2688 |
| 100 | 6 | cmb0607.0 | 7,077,663 | 0.6917 | 3 | 0.0167 | 0.4221 |
| 101 | 6 | cmb0607.8 | 7,841,654 | 0.8500 | 2 | 0.0000 | 0.2225 |
| 102 | 6 | id6005608 | 8,725,792 | 0.7167 | 2 | 0.0000 | 0.3236 |
| 103 | 6 | cmb0610.0 | 10,084,821 | 0.8167 | 2 | 0.0000 | 0.2546 |
| 104 | 6 | id6008118 | 13,653,907 | 0.7333 | 2 | 0.0000 | 0.3146 |
| 105 | 6 | cmb0614.6 | 14,670,163 | 0.7500 | 3 | 0.0000 | 0.3212 |
| 106 | 6 | id6009699 | 18,066,599 | 0.7333 | 3 | 0.0000 | 0.3319 |
| 107 | 6 | cmb0618.2 | 18,278,072 | 0.7000 | 2 | 0.0000 | 0.3318 |
| 108 | 6 | id6011555 | 23,136,975 | 0.7917 | 2 | 0.0167 | 0.2755 |
| 109 | 6 | cmb0625.3 | 25,364,319 | 0.7667 | 2 | 0.0000 | 0.2938 |
| 110 | 6 | id6015530 | 27,381,493 | 0.8417 | 3 | 0.0500 | 0.2424 |
| 111 | 6 | cmb0629.3 | 29,386,973 | 0.7500 | 2 | 0.0000 | 0.3047 |
| 112 | 6 | id6016941 | 31,686,494 | 0.7667 | 2 | 0.0000 | 0.2938 |
| 113 | 7 | cmb0700.1 | 122,298 | 0.8000 | 2 | 0.0000 | 0.2688 |
| 114 | 7 | ud7000187 | 2,563,993 | 0.5500 | 2 | 0.0000 | 0.3725 |
| 115 | 7 | cmb0703.2 | 3,292,230 | 0.6167 | 2 | 0.0000 | 0.3610 |
| 116 | 7 | ad07001853 | 4,265,929 | 0.7333 | 2 | 0.0000 | 0.3146 |
| 117 | 7 | id7001155 | 7,019,781 | 0.5167 | 2 | 0.0000 | 0.3747 |
| 118 | 7 | id7001998 | 11,588,122 | 0.5333 | 2 | 0.0000 | 0.3739 |
| 119 | 7 | id7002392 | 15,667,894 | 0.5667 | 2 | 0.0000 | 0.3705 |
| 120 | 7 | cmb0718.0 | 18,017,078 | 0.5833 | 2 | 0.0000 | 0.3680 |
| 121 | 7 | id7003072 | 20,321,276 | 0.6000 | 2 | 0.0000 | 0.3648 |
| 122 | 7 | cmb0723.0 | 23,014,905 | 0.7667 | 2 | 0.0000 | 0.2938 |
| 123 | 7 | SLG7-GC | 25,326,292 | 0.6667 | 2 | 0.0000 | 0.3457 |
| 124 | 7 | id7004645 | 25,682,560 | 0.6333 | 2 | 0.0000 | 0.3566 |
| 125 | 7 | cmb0727.0 | 27,003,411 | 0.7333 | 2 | 0.0000 | 0.3146 |
| 126 | 7 | cmb0728.5 | 28,576,878 | 0.5333 | 2 | 0.0000 | 0.3739 |
| 127 | 7 | id7006027 | 29,931,834 | 0.5667 | 2 | 0.0000 | 0.3705 |
| 128 | 7 | cmb0730.3 | 30,325,349 | 0.6667 | 2 | 0.0000 | 0.3457 |
| 129 | 8 | id8000140 | 416,700 | 0.7333 | 3 | 0.0000 | 0.3319 |
| 130 | 8 | cmb0801.3 | 1,318,681 | 0.4833 | 3 | 0.0000 | 0.4610 |
| 131 | 8 | cmb0802.8 | 2,855,479 | 0.7333 | 2 | 0.0000 | 0.3146 |
| 132 | 8 | id8001426 | 4,363,186 | 0.7000 | 2 | 0.0000 | 0.3318 |
| 133 | 8 | cmb0805.6 | 5,648,540 | 0.8167 | 2 | 0.0000 | 0.2546 |
| 134 | 8 | wd8001250 | 8,424,668 | 0.7667 | 2 | 0.0000 | 0.2938 |
| 135 | 8 | id8003584 | 11,798,820 | 0.7750 | 2 | 0.0167 | 0.2879 |
| 136 | 8 | id8004111 | 15,352,572 | 0.7500 | 2 | 0.0000 | 0.3047 |
| 137 | 8 | id8005186 | 19,436,398 | 0.5333 | 2 | 0.0000 | 0.3739 |
| 138 | 8 | ae08007378 | 21,503,195 | 0.8167 | 2 | 0.0000 | 0.2546 |
| 139 | 8 | id8006751 | 23,740,826 | 0.6000 | 2 | 0.0000 | 0.3648 |
| 140 | 8 | cmb0824.7 | 24,799,130 | 0.5167 | 2 | 0.0000 | 0.3747 |
| 141 | 8 | GW8-AG | 26,590,280 | 0.6333 | 2 | 0.0000 | 0.3566 |
| 142 | 8 | id8007764 | 27,921,569 | 0.6000 | 2 | 0.0000 | 0.3648 |
| 143 | 9 | id9000045 | 438,538 | 0.7333 | 2 | 0.0000 | 0.3146 |
| 144 | 9 | id9000884 | 4,288,667 | 0.6333 | 2 | 0.0000 | 0.3566 |
| 145 | 9 | cmb0907.2 | 7,214,648 | 0.5667 | 2 | 0.0000 | 0.3705 |
| 146 | 9 | id9002419 | 8,513,936 | 0.7000 | 2 | 0.0000 | 0.3318 |
| 147 | 9 | id9003183 | 12,505,826 | 0.6333 | 2 | 0.0000 | 0.3566 |
| 148 | 9 | cmb0914.4 | 14,468,502 | 0.7667 | 2 | 0.0000 | 0.2938 |
| 149 | 9 | id9004072 | 15,394,586 | 0.5667 | 2 | 0.0000 | 0.3705 |
| 150 | 9 | ae09005437 | 17,509,141 | 0.7833 | 2 | 0.0000 | 0.2818 |
| 151 | 9 | id9006953 | 20,044,954 | 0.5333 | 2 | 0.0000 | 0.3739 |
| 152 | 9 | TAC1-CT | 21,615,845 | 0.6333 | 2 | 0.0000 | 0.3566 |
| 153 | 9 | id9007784 | 23,484,844 | 0.6167 | 2 | 0.0000 | 0.3610 |
| 154 | 10 | id10000113 | 587,669 | 0.5167 | 3 | 0.0333 | 0.5374 |
| 155 | 10 | ud10000265 | 3,982,886 | 0.7833 | 2 | 0.0000 | 0.2818 |
| 156 | 10 | id10002069 | 6,305,120 | 0.5000 | 2 | 0.0000 | 0.3750 |
| 157 | 10 | id10002842 | 10,730,617 | 0.7250 | 2 | 0.0167 | 0.3192 |
| 158 | 10 | id10003706 | 14,754,458 | 0.6167 | 2 | 0.0000 | 0.3610 |
| 159 | 10 | cmb1016.4 | 16,412,329 | 0.7833 | 3 | 0.0000 | 0.2967 |
| 160 | 10 | cmb1018.3 | 18,307,117 | 0.5500 | 2 | 0.0000 | 0.3725 |
| 161 | 10 | wd10003790 | 19,917,460 | 0.7833 | 2 | 0.0000 | 0.2818 |
| 162 | 10 | ah10001182 | 21,598,545 | 0.6333 | 2 | 0.0000 | 0.3566 |
| 163 | 10 | id10007384 | 23,690,908 | 0.6333 | 2 | 0.0000 | 0.3566 |
| 164 | 11 | id11000131 | 680,665 | 0.6750 | 2 | 0.0167 | 0.3425 |
| 165 | 11 | cmb1102.6 | 2,669,824 | 0.8167 | 2 | 0.0000 | 0.2546 |
| 166 | 11 | cmb1104.6 | 4,667,865 | 0.6083 | 3 | 0.0167 | 0.3847 |
| 167 | 11 | id11002336 | 5,628,396 | 0.6167 | 2 | 0.0000 | 0.3610 |
| 168 | 11 | cmb1107.1 | 7,109,268 | 0.7167 | 2 | 0.0000 | 0.3236 |
| 169 | 11 | wd11000649 | 9,670,517 | 0.6250 | 3 | 0.0167 | 0.4443 |
| 170 | 11 | cmb1109.8 | 9,835,830 | 0.6667 | 2 | 0.0000 | 0.3457 |
| 171 | 11 | id11004341 | 15,215,162 | 0.6000 | 2 | 0.0000 | 0.3648 |
| 172 | 11 | cmb1119.1 | 19,164,872 | 0.7333 | 2 | 0.0000 | 0.3146 |
| 173 | 11 | id11006897 | 21,212,729 | 0.5000 | 2 | 0.0000 | 0.3750 |
| 174 | 11 | cmb1121.9 | 21,935,946 | 0.4583 | 3 | 0.0833 | 0.4948 |
| 175 | 11 | id11008929 | 25,372,661 | 0.6667 | 2 | 0.0000 | 0.3457 |
| 176 | 11 | cmb1127.4 | 27,413,918 | 0.6333 | 2 | 0.0000 | 0.3566 |
| 177 | 11 | id11011607 | 31,063,738 | 0.5167 | 3 | 0.0000 | 0.4894 |
| 178 | 12 | id12000076 | 264,373 | 0.7667 | 2 | 0.0000 | 0.2938 |
| 179 | 12 | cmb1202.4 | 2,420,416 | 0.7333 | 2 | 0.0000 | 0.3146 |
| 180 | 12 | P0523 | 3,024,346 | 0.7000 | 2 | 0.0000 | 0.3318 |
| 181 | 12 | id12002113 | 4,662,617 | 0.7667 | 2 | 0.0000 | 0.2938 |
| 182 | 12 | cmb1207.0 | 7,038,537 | 0.5750 | 3 | 0.0167 | 0.4955 |
| 183 | 12 | id12003700 | 9,135,012 | 0.6833 | 2 | 0.0000 | 0.3391 |
| 184 | 12 | id12005212 | 14,646,923 | 0.8333 | 2 | 0.0000 | 0.2392 |
| 185 | 12 | id12006155 | 18,468,907 | 0.5167 | 2 | 0.0000 | 0.3747 |
| 186 | 12 | cmb1221.7 | 21,760,477 | 0.8500 | 3 | 0.0000 | 0.2333 |
| 187 | 12 | id12007742 | 23,031,189 | 0.5500 | 2 | 0.0000 | 0.3725 |
| 188 | 12 | cmb1224.0 | 24,056,822 | 0.5333 | 2 | 0.0000 | 0.3739 |
| 189 | 12 | cmb1226.0 | 26,032,801 | 0.6833 | 2 | 0.0000 | 0.3391 |
| 190 | 12 | id12010130 | 27,582,487 | 0.5667 | 2 | 0.0000 | 0.3705 |
|  |  | Mean | - | 0.6816 | 2.12 | 0.0020 | 0.3328 |

*^a^*MAF, major allele frequency.
*^b^*PIC, polymorphic information content.

| **Supplementary Table 6.** Average mean performance and genetic variability of 13 agronomic and grain quality traits of 30 traditional Basmati varieties. | | | | | | | | | | | | | | | | | |
| --- | --- | --- | --- | --- | --- | --- | --- | --- | --- | --- | --- | --- | --- | --- | --- | --- | --- |
| **No.** | **Name** | **Subgroup** | **Cluster** | **DH** | **LW** | **DM** | **CL (cm)** | **CN** | **CD (mm)** | **GL (mm)** | **GW (mm)** | **L/W ratio** | **KGW (g)** | **PL (cm)** | **SFC** | **AC (%)** |  |
| 1 | Basmati Dhan | *Aromatic* | II | 111 | N/A | 141 | N/A | N/A | N/A | 5.5 | 2.4 | 2.29 | 14 | N/A | 3 | 24 |  |
| 2 | Dhera Dun Basmati | *Aus* | I | 73 | 1.1 | 103 | 92 | 7 | 4 | 8.5 | 2.8 | 3.04 | 18 | 24 | 1 | 28 |  |
| 3 | Basmati Nahan 381 | *Aromatic* | I | 84 | 1.6 | 114 | 134 | 19 | 5 | 9.6 | 2.9 | 3.31 | 26 | 30 | 3 | 25 |  |
| 4 | Basmati Sufaid 100 | *Aromatic* | I | 84 | 1.2 | 114 | 143 | 20 | 4 | 9.6 | 2.4 | 4 | 24 | 32 | 3 | 24 |  |
| 5 | Basmati 140 | *Aus* | I | 68 | 1 | 98 | 105 | 25 | 4 | 8.8 | 3.2 | 2.75 | 29 | 22 | 3 | 24 |  |
| 6 | Basmati 370 | *Aromatic* | I | 84 | 1.1 | 114 | 134 | 22 | 4 | 9.8 | 2.3 | 4.26 | 23 | 32 | 3 | 19 |  |
| 7 | Basmati 372 | *Aromatic* | I | 84 | 1.1 | 114 | 122 | 20 | 4 | 10 | 2.3 | 4.35 | 24 | 30 | 3 | 18 |  |
| 8 | Basmati 377 | *Aromatic* | I | 84 | 1 | 114 | 129 | 19 | 4 | 9.7 | 2.4 | 4.04 | 23 | 32 | 3 | 19 |  |
| 9 | Dera Duni Basmati 321 | *Aromatic* | I | 84 | 1.4 | 114 | 143 | 15 | 5 | 9.4 | 2.7 | 3.48 | 25 | 30 | 3 | 21 |  |
| 10 | Kamoh Basmati 392 | *Aromatic* | I | 84 | 1.3 | 114 | 130 | 18 | 4 | 10 | 2.2 | 4.55 | 24 | 31 | 3 | 20 |  |
| 11 | Sathi Basmati | *Aromatic* | I | 84 | 1.1 | 114 | 115 | 19 | 4 | 10.7 | 2.3 | 4.65 | 26 | 30 | 3 | 20 |  |
| 12 | Basmati Sal | *Aus* | I | 84 | 1.1 | 114 | 78 | 11 | 5 | 9.3 | 3.3 | 2.82 | 16 | 22 | 3 | N/A |  |
| 13 | Basmati Kunar | *Aromatic* | I | 88 | 1 | 118 | 115 | 21 | 4 | 9.4 | 2.3 | 4.09 | 22 | 20 | 3 | 20 |  |
| 14 | Basmati Kunduz | *Indica* | I | 78 | 1 | 108 | 93 | 29 | 5 | 8.1 | 2.9 | 2.79 | 24 | 20 | 3 | 24 |  |
| 15 | Basmati Anpjhutte | *Aromatic* | I | 92 | 1.2 | 122 | 113 | 17 | 4 | 8 | 2.3 | 3.48 | 17 | 24 | 3 | 17 |  |
| 16 | Basmati Ola | *Aromatic* | II | 124 | N/A | 154 | N/A | N/A | N/A | 8 | 2.5 | 3.2 | 19 | N/A | 3 | N/A |  |
| 17 | Basmati Lamo | *Aromatic* | II | 134 | 1.2 | 164 | 142 | 15 | 5 | 8 | 2.5 | 3.2 | 18 | 24 | 3 | 18 |  |
| 18 | Basmati Masino | *Indica* | II | 102 | 1.1 | 132 | 153 | 15 | 5 | 6.6 | 3.1 | 2.13 | 18 | 24 | 3 | 26 |  |
| 19 | Basmati Nokhi | *Indica* | II | 122 | 1.2 | 152 | 123 | 19 | 4 | 8.7 | 2.7 | 3.22 | 19 | 28 | 3 | 25 |  |
| 20 | Basmati Pahade | *Aromatic* | II | 103 | 1.1 | 133 | 143 | 18 | 5 | 6.8 | 2.8 | 2.43 | 15 | 26 | 3 | 19 |  |
| 21 | Basmati Red | *Aromatic* | II | 124 | 1.1 | 154 | 141 | 13 | 5 | 8.7 | 2.3 | 3.78 | 20 | 29 | 3 | N/A |  |
| 22 | Basmati White | *Aromatic* | I | 99 | N/A | 129 | N/A | N/A | N/A | 8.1 | 2.2 | 3.68 | 18 | N/A | 3 | N/A |  |
| 23 | Basmati Uzarka | *Aromatic* | II | 130 | 1.3 | 160 | 152 | 14 | 5 | 8.4 | 2.5 | 3.36 | 17 | 26 | 3 | 19 |  |
| 24 | Kalo Basmati | *Aromatic* | I | 88 | 0.8 | 118 | 121 | 14 | 4 | 8.5 | 2.4 | 3.54 | 18 | 22 | 3 | 17 |  |
| 25 | Rato Basmati | *Aromatic* | II | 118 | 1.1 | 148 | 158 | 19 | 4 | 8.6 | 2.5 | 3.44 | 20 | 27 | 3 | N/A |  |
| 26 | Basmati Mwea | *Aromatic* | I | 92 | 1.1 | 122 | 112 | 12 | 4 | 9.6 | 2.3 | 4.17 | 22 | 26 | 1 | N/A |  |
| 27 | Dahrdun Basmati | *Indica* | II | 108 | 1.3 | 138 | 161 | 16 | 6 | 10.8 | 2.4 | 4.5 | 25 | 28 | 1 | N/A |  |
| 28 | Basmatiya | *Aus* | I | 92 | N/A | 122 | N/A | N/A | N/A | 8.6 | 2.5 | 3.44 | 18 | N/A | 3 | N/A |  |
| 29 | Pakistani Basmati | *Aromatic* | I | 96 | 1.2 | 126 | 130 | 18 | 5 | 10.5 | 2.2 | 4.77 | 23 | 26 | 1 | N/A |  |
| 30 | Karnal Basmati | *Aromatic* | I | 102 | 1.2 | 132 | 105 | 23 | 5 | 11 | 2.4 | 4.58 | 23 | 27 | 3 | N/A |  |
| Average | | | | 96.67 | 1.15 | 126.67 | 126.42 | 17.62 | 4.5 | 8.91 | 2.53 | 3.58 | 20.93 | 26.62 | 2.73 | 21.35 |  |
| Minimum | | | | 68 | 0.8 | 98 | 78 | 7 | 4 | 5.5 | 2.2 | 2.1 | 14 | 20 | 1 | 17 |  |
| Maximum | | | | 134 | 1.6 | 164 | 161 | 29 | 6 | 11 | 3.3 | 4.8 | 29 | 32 | 3 | 28 |  |
| Standard deviation | | | | 17.25 | 0.15 | 17.25 | 20.81 | 4.49 | 0.57 | 1.23 | 0.3 | 0.71 | 3.68 | 3.63 | 0.68 | 3.23 |  |
| Coefficient of variation (%) | | | | 17.84 | 13.04 | 13.62 | 16.46 | 25.49 | 12.71 | 13.84 | 11.72 | 19.98 | 17.57 | 13.63 | 24.87 | 15.12 |  |
| *Traits: DH, days to heading; LW, leaf width; DM, days to maturity; CL, culm length; CN, culm Number; CD, culm diameter; GL, grain length; GW, grain width; L/W ratio, length-to-width ratio of grain; KGW, 1000 grain weight, PL, panicle length; SFC, spikelet fertility count; AC, amylose content. Passport data was accessed on November 8^th^, 2018 from the Genesys public database. | | | | | | | | | | | | | | | | | |
|  |  |  |  |  |  |  |  |  |  |  |  |  |  |  |  |  |  |
|  |  |  |  |  |  |  |  |  |  |  |  |  |  |  |  |  |  |

**Supplementary Table 7.** Comparison of agronomic and grain quality traits between *aromatic* and *indica/aus* Basmati groups

| **Rice groups** | **Traits*^a^*** | | | | | | | | | | | | |
| --- | --- | --- | --- | --- | --- | --- | --- | --- | --- | --- | --- | --- | --- |
|  | **DH** | **LW (cm)** | **DM** | **CL (cm)** | **CN** | **CD (mm)** | **GL (mm)** | **GW (mm)** | **L/W ratio** | **KGW (g)** | **PL (cm)** | **SFC** | **AC (%)** |
| ***Aromatic*** | 98.77 ± 16.67 | 1.16 ± 0.16 | 128.77 ± 16.67 | 130.63 ± 14.32 | 17.68 ± 3.01 | 4.42 ± 0.49 | 8.99 ± 1.27 | 2.41 ± 0.18 | 3.75 ± 0.65 | 20.95 ± 3.44 | 27.57 ± 3.41 | 2.81 ± 0.57 | 20 ± 2.42 |
| ***Indica*/*Aus*** | 90.87 ± 17.47 | 1.11 ± 0.10 | 120.87 ± 17.47 | 115 ± 29.53 | 17.42 ± 7.09 | 4.71 ± 0.70 | 8.67 ± 1.10 | 2.86 ± 0.30 | 3.08 ± 0.65 | 20.87 ± 4.26 | 24 ± 2.83 | 2.5 ± 0.87 | 25.40 ± 1.50 |
| **Significance** | NS | NS | NS | NS | NS | NS | NS | NS | * | NS | * | NS | ** |

*^a^*Traits: DH, days to heading; LW, leaf width; DM, days to maturity; CL, culm length; CN, culm number; CD, culm diameter; GL, grain length; GW, grain width; L/W ratio, length-to-width ratio of grain; KGW, 1,000 grain weight; PL, panicle length; SFC, spikelet fertility count; AC, amylose content. Data represent mean ± standard error (SE) of three replications. Asterisks indicate the level of significance (**P* = 0.05; ***P* = 0.01). NS, non-significant.

**Supplementary Table 8.** Genes involved in aromatic biosynthesis process based on biological process and molecular functional annotation

| **GO Id** | **Gene name** | **Description** | **Allele types*^a^*** | | | |
| --- | --- | --- | --- | --- | --- | --- |
|  |  |  | **Nipponbare** | **Basmati 370** | **Dahrdun Basmati** | **Rato Basmati** |
| GO:0044237 | *Os06g0281300* | Similar to O-methyltransferase ZRP4 | R | A 1 | A 2 | A 1 |
| GO:0044237 | *Os08g0290700* | Winged helix repressor DNA-binding domain containing protein | R | A 1 | A 2 | A 1 |
| GO:0044237 | *Os08g0454500* | Similar to Flavonoid 7-O-methyltransferase | R | A 1 | A 2 | A 1 |
| GO:0044237 | *Os09g0344500* | Acetylserotonin O-methyltransferase 1 | R | A 1 | A 2 | A 1 |
| GO:0044237 | *Os10g0118000* | Acetylserotonin O-methyltransferase 3 | R | A 1 | A 2 | A 1 |
| GO:0044237 | *Os10g0118200* | Acetylserotonin O-methyltransferase 2 | R | A 1 | A 2 | A 1 |
| GO:0044237 | *Os12g0199500* | Similar to N-methyltransferase | R | A 1 | A 2 | A 1 |
| GO:0044237 | *Os12g0240900* | Naringenin 7-O-methyltransferase | R | A 1 | R | A 1 |
| GO:0003674 | *Os08g0424500* | betaine-aldehyde dehydrogenase activity, *Badh2* | R | A 1 | A 2 | A 1 |

*^a^*Allele types: R, Reference genome allele; A1, Alternative allele 1; A2, Alternative allele 2. The allele type was determined using non-synonymous SNPs and SNPs in UTR region.
